# Shotgun metagenomic profiling of the fecal microbiome in *Lamp2* knockout mice reveals limited genotype-associated differences under standard housing

**DOI:** 10.1371/journal.pone.0357009

**Published:** 2026-09-18

**Authors:** Maria Emfietzoglou, Maria Anna Bantounou, Shewa Osmani, Toshio Narimatsu, Konstantinos G. Baroutis, Alexandra Varsamidaki, Dimitra Skondra, Dimitris Papaconstantinou, Panagiotis Theodossiadis, Irini Chatziralli, Joan W. Miller, Demetrios G. Vavvas

**Affiliations:** 1 Angiogenesis Laboratory, Ines and Frederick Yeatts Retina Research Laboratory, Massachusetts Eye and Ear, Department of Ophthalmology, Harvard Medical School, Boston, Massachusetts, United States of America; 2 NYU Langone Health, NYU Grossman School of Medicine, New York, New York, United States of America; 3 Department of Ophthalmology, National and Kapodistrian University of Athens, Athens, Greece; Florida A&M University: Florida Agricultural and Mechanical University, UNITED STATES OF AMERICA

## Abstract

**Background:**

Lysosomal pathways influence host–microbe interactions, but the microbiome consequences of lysosomal dysfunction remain incompletely defined. LAMP2 is required for autophagosome–lysosome fusion, and pathogenic variants in *LAMP2* cause Danon disease. Whether *Lamp2* loss alters the gut microbiome in vivo has not been systematically evaluated using methods that profile both taxonomic composition and microbial functional potential, such as shotgun metagenomics.

**Methods:**

We performed shotgun metagenomic sequencing on 50 fecal samples from male *Lamp2* knockout (*Lamp2*KO) mice and wild-type (WT) littermates sampled at 3, 6, 9, and 12 months under single-genotype cages or mixed-genotype cohousing. Two low-depth libraries (<3 × 10^5^ classified genus-level reads) were excluded from primary inference (primary set: n = 48). We analyzed genus-level alpha diversity, beta diversity, and differential abundance using compositional, cage-aware mixed-effects models and cage-blocked permutation testing. We analyzed functional pathway profiles using copies-per-million abundances with centered-log-ratio transformation and mixed-effects modeling. We controlled multiple testing using the Benjamini–Hochberg false discovery rate.

**Results:**

In the primary set (48 samples from 27 cages), *Lamp2*KO and WT mice showed similar genus-level alpha diversity and overall community composition (PERMANOVA using Aitchison and Bray–Curtis distances). Primary mixed-effects models detected no genera with differential abundance after false discovery rate correction. Taxonomic profiles were broadly similar between genotypes and were dominated by Bacteroidota and Bacillota. Exploratory within-cage (paired) analyses identified consistent directional differences in a small set of genera, but these signals were not supported by the primary mixed-effects models. Functional pathway profiles were similar between genotypes; one pathway (dTDP-β-L-rhamnose biosynthesis) showed an exploratory association (FDR q < 0.10) within the 50 most abundant pathways.

**Conclusions:**

In this controlled mouse cohort, we did not detect robust, cage-independent shifts in fecal microbiome composition or inferred functional pathway profiles associated with *Lamp2* deficiency under standard SPF husbandry and chow; given the sample size, smaller or compartment-specific effects cannot be excluded.

## Introduction

The gut microbiome shapes host metabolism, immune homeostasis, and intestinal barrier integrity [1]. Variation in microbial community structure and functional potential has been linked to a wide range of systemic disorders, including metabolic and inflammatory disease, neurodegeneration, and ocular conditions [2]. In mouse models, host genetic and cellular pathways can shape microbial ecology, but genotype-associated microbiome differences are often modest and can be obscured by environmental variation; studies must therefore distinguish host-intrinsic effects from shared exposures [3].

Autophagy and lysosomal pathways regulate host–microbe interactions by maintaining epithelial homeostasis, modulating immune signaling, clearing microbes, and shaping nutrient availability in the gut lumen [4]. Disruption of autophagy-related genes can shift gut microbial composition in several genetic models, particularly under inflammatory or metabolic stress [4,5]. These findings support a mechanistic link between lysosomal function and microbial ecology, but they do not establish whether loss of a specific lysosomal component produces a reproducible microbiome signature under standard conditions.

Lysosome-associated membrane protein 2 (LAMP2) is a core lysosomal membrane protein required for autophagosome–lysosome fusion and efficient autophagic degradation [6]. Pathogenic variants in *LAMP****2*** cause Danon disease, a rare X-linked lysosomal disorder characterized by cardiomyopathy and skeletal myopathy, with multisystem involvement that can include hepatic and gastrointestinal manifestations as well as retinal disease [7,8]. *Lamp2*-deficient mouse models recapitulate key features of the human disorder and are widely used to study lysosome-dependent processes across tissues, including heart and retina [9–11]. Given the central role of lysosomal function in epithelial and immune homeostasis, *Lamp2* loss could plausibly alter intestinal physiology and host–microbe interactions [12]. However, the gut microbiome has not been systematically characterized in *Lamp2* deficiency. It remains unclear whether loss of *Lamp2* produces detectable changes in fecal microbial diversity, taxonomic composition, or functional capacity. Establishing whether *Lamp2* deficiency perturbs the gut microbiome may also help interpret systemic contributors to Danon cardiomyopathy and retinopathy, given emerging evidence that gut microbial communities and metabolites can influence cardiovascular and retinal phenotypes (the gut–heart and gut–retina axes).

We addressed this question using shotgun metagenomic sequencing rather than marker-gene (16S rRNA) profiling. Whereas amplicon approaches survey a single marker gene—typically limiting resolution to the genus level and introducing primer and 16S copy-number biases—shotgun metagenomics sequences total community DNA, supporting higher taxonomic resolution (to the species level in this study) and, importantly, direct inference of microbial functional potential from gene content [13]. This added resolution and functional dimension make shotgun metagenomics well suited to detecting subtle, host-genotype–associated shifts in both community composition and metabolic capacity, and they raise the evidentiary bar for concluding such shifts are absent: a higher-resolution, function-aware method that nonetheless detects minimal differences provides stronger evidence against a robust microbiome effect than a lower-resolution survey would.

Here, we profiled fecal microbiomes from male *Lamp2* knockout (*Lamp2*KO) mice and wild-type littermate controls sampled across four age groups and two cage configurations: **single-genotype cages** and **mixed-genotype cohousing.** We tested for genotype-associated differences in alpha diversity, beta diversity, taxonomic composition, and inferred functional pathways using compositional data analysis and cage-aware statistical models. Our goal was to establish a conservative baseline for fecal community structure in *Lamp2*KO mice under standard housing conditions.

## Methods

### Animals

*Lamp2* knockout (*Lamp2* − /Y) mice were originally generated in the laboratory of Prof. Paul Saftig (Kiel University, Germany) and provided by Shiga University of Medical Science (Shiga, Japan). The line was backcrossed onto a C57BL/6J background. Experimental animals were generated by breeding heterozygous females (*Lamp2* + /−) with wild type (*Lamp2* + /Y) males, yielding male knockout (*Lamp2* − /Y; hereafter *Lamp2*KO) and wild-type male littermates (*Lamp2* + /Y; hereafter WT). Because *Lamp2* is X-linked, hemizygous males (*Lamp2* − /Y) are uniformly null, whereas heterozygous females (*Lamp2 + /−*) are mosaic for X-inactivation and show variable phenotypic penetrance, paralleling the more severe and uniform presentation of male patients with Danon disease. We therefore studied only male mice to model a uniform loss-of-function genotype; sex-based and sex-by-genotype comparisons were consequently not possible by design. Mice were maintained in a specific pathogen–free (SPF) barrier facility at Massachusetts Eye and Ear under a 12-hour light/dark cycle, with standard chow and water provided ad libitum. Male mice were sampled at 3, 6, 9, and 12 months of age and were housed either in single-genotype cages (WT-only or *Lamp2*KO-only) or in mixed-genotype cages containing both genotypes (Supplementary S2 Table in S1 File). All animal procedures were approved by the Massachusetts General Brigham Institutional Animal Care and Use Committee (IACUC; protocol 2021N000118). All procedures adhered to the ARVO Statement for the Use of Animals in Ophthalmic and Vision Research. This study did not involve human participants or human-derived samples; therefore, institutional review board approval and informed consent were not applicable.

### Sample collection

Fecal samples were collected using Transnetyx Microbiome kits containing pre-barcoded tubes (Transnetyx, Cordova, TN, USA) prefilled with Norgen Stool Nucleic Acid Preservative (Norgen Biotek, Thorold, ON, Canada). At approximately 2:00 PM, each mouse was briefly placed in an autoclaved cage without bedding until it produced 1–2 fresh pellets. Pellets were transferred into the preservative using a sterile pipette tip (one per mouse), and the tip was discarded after use. Samples were shipped at room temperature to Transnetyx for downstream processing.

### DNA extraction, library preparation, and sequencing

Shotgun metagenomic sequencing randomly sequences DNA fragments from all organisms present in a sample, enabling taxonomic profiling beyond marker-gene approaches and inference of microbial functional potential from gene content. Genomic DNA was extracted using the DNeasy 96 PowerSoil Pro Kit on the QIAcube HT automated platform (Qiagen, Germantown, MD, USA), following the manufacturer’s protocol. DNA quality was assessed prior to library preparation. Sequencing libraries were generated with the KAPA HyperPlus Kit (Roche Sequencing, Pleasanton, CA, USA) using unique dual-indexed adapters to reduce index hopping and cross-sample misassignment. Libraries were quality-checked and sequenced on the Illumina NextSeq 2000 platform (Illumina, San Diego, CA, USA) with paired-end reads (2 × 150 bp).

### Bioinformatic analysis

Raw FASTQ reads were processed using the One Codex platform (One Codex, San Francisco, CA, USA). Reads were classified using the One Codex k-mer–based taxonomic classifier against the One Codex reference database (2025 H1 release). At the time of analysis (January 2026), the platform applied standard preprocessing steps, including quality filtering and identification of host-associated and non-specific reads, prior to microbial profiling. Taxonomic abundance tables were exported from filtered One Codex outputs at the genus level for downstream statistical analyses. Although shotgun profiling can resolve taxa below the genus level (e.g., to the species level), genus-level tables were used for inferential analyses to reduce sparsity and classifier-level misassignment, which are pronounced for low-biomass features in fecal shotgun data and which inflate variance in differential-abundance testing. Relative-abundance summaries at the phylum, genus, and species levels were used for descriptive taxonomic plots. Functional pathway profiles were generated by One Codex using its internal functional annotation pipeline (MetaCyc pathway definitions) and exported as copies-per-million (CPM) tables for downstream analysis.

### Statistical analysis

Statistical analyses were performed in R. Sequencing batch and cage structure were incorporated into inferential analyses to account for technical variation and shared housing, respectively. Unless otherwise specified, tests were two-sided and multiple testing was controlled using the Benjamini–Hochberg false discovery rate (FDR).

### Quality control and primary analysis set

Sequencing and classification quality metrics (total reads, mapped reads, percent mapped, percent specific microbial, percent unclassified, and percent host/non-specific) were obtained from One Codex outputs and summarized descriptively (see Results). “Host/non-specific” reads denote sequences assigned to host genomes or to non-unique microbial classifications by the pipeline. We included this metric as a covariate in all community analyses and additionally examined it as a candidate biological signal (see Results and Discussion). Transnetyx performed vendor-level quality control and re-sequenced libraries that did not reach their target depth; all vendor controls passed QC. For inferential analyses, we prespecified a minimum depth threshold of 3 × 10^5^ classified genus-level reads; two libraries below this threshold were excluded from the primary analysis set. To confirm that excluding these two libraries did not influence our conclusions, we repeated all genus-level analyses on the full cohort (n = 50); results were concordant with the primary analysis set and are reported in Supplementary Table S8 in S1 File.

### Data preparation and compositional transformations

Per-sample sequencing depth was defined as the sum of classified genus-level reads and was included as a covariate (log10-transformed) in regression models. To reduce sparsity, genus-level analyses were restricted to genera detected (non-zero counts) in ≥10% of samples in the primary analysis set (i.e., present in at least 10% of samples across the cohort). Because metagenomic abundance profiles are compositional (only relative information is meaningful), genus- and pathway-level differential abundance and Aitchison distance analyses were performed using centered-log-ratio (CLR) transformed abundances. To accommodate zeros, a pseudocount of 0.5 was added to feature counts prior to CLR transformation. The CLR transformation computes log-abundance relative to the sample’s geometric mean across features; Euclidean distances in CLR space correspond to Aitchison distances.

### Alpha diversity

Genus-level alpha diversity (within-sample diversity) was quantified using three metrics: Shannon diversity (accounts for richness and evenness), Simpson diversity (1 − D; higher values indicate greater evenness), and observed genus richness (the number of genera with non-zero counts after prevalence filtering). Alpha diversity metrics were analyzed using linear mixed-effects models with cage included as a random intercept to account for shared housing. Fixed effects included genotype, housing condition, age group, sequencing batch, and log10 sequencing depth.

### Beta diversity

Between-sample community dissimilarity (beta diversity) was evaluated using Aitchison distance (primary; Euclidean distance on CLR-transformed genus abundances) and Bray–Curtis distance on relative abundances (secondary). Community structure was visualized using principal coordinate analysis (PCoA), which projects distance relationships into a lower-dimensional space. Group differences in community composition were tested using permutational multivariate analysis of variance (PERMANOVA; adonis2, vegan package), a permutation-based test of whether multivariate community composition differs across groups. PERMANOVA used 999 permutations constrained within cages (strata = cage) and marginal sums of squares (by = “margin”). The primary model was distance ~ genotype + housing condition + age group + sequencing batch. Homogeneity of multivariate dispersion was assessed using PERMDISP (betadisper), which tests whether within-group dispersion differs (a potential confound for PERMANOVA). As a sensitivity analysis, we repeated PERMANOVA including a genotype-by-housing interaction term.

### Differential abundance

Genus-level differential abundance was tested using linear mixed-effects models applied to CLR-transformed abundances, with cage modeled as a random intercept. Fixed effects included genotype (primary contrast), housing condition, age group, sequencing batch, log10 sequencing depth, and host/non-specific read fraction where available. P-values for genotype effects were adjusted across genera using Benjamini–Hochberg FDR.

### Paired-cage analysis

In mixed-genotype cages, we performed within-cage comparisons by computing differences in CLR abundance (*Lamp2*KO − WT) for each genus using cage-level means and evaluating whether the median difference across cages differed from zero (Wilcoxon signed-rank test). P-values were adjusted using Benjamini–Hochberg FDR. This paired analysis was treated as exploratory because it conditions on a subset of cages and reduces the effective sample size. Genera highlighted by this exploratory analysis were additionally screened for laboratory or reagent contamination by cross-referencing against published common-contaminant genera [14], examining their relative abundance, and testing whether their relative abundance was inversely associated with sequencing depth (a biomass-based frequency diagnostic); formal decontam-based classification [15] was not applied because the dataset did not include negative (blank) controls or DNA quantitation.

### Taxonomic composition

Taxonomic profiles were summarized descriptively using One Codex relative abundance outputs. For each taxonomic rank (phylum, genus, and species), we summarized cohort-level mean relative abundances and focused plots on the 10 most abundant taxa, grouping remaining taxa as “Other.”

### Functional analysis

Functional profiling used pathway-level CPM tables generated by the One Codex functional analysis pipeline. For cohort-level comparisons, we analyzed the 50 most abundant pathways across the cohort — the most stably quantified features and the standard set returned by the platform’s functional export — to focus on well-measured pathways and limit multiple testing. To confirm this focus did not omit genotype-associated pathways, we additionally analyzed all detected pathways (n = 214; CLR-transformed pathway abundances) using the same cage-aware mixed-effects model; complete per-pathway results are provided in the deposited project data. Pathway abundances were CLR-transformed and analyzed using Aitchison distances, PCoA, and cage-blocked PERMANOVA as described above. To test genotype-associated differences at the individual pathway level, each pathway was analyzed using a linear mixed-effects model with cage included as a random intercept. Fixed effects included genotype, housing condition, age group, sequencing batch, log10 sequencing depth, and host/non-specific read fraction where available. P-values for genotype effects were adjusted across pathways using Benjamini–Hochberg FDR.

### Statistical power

To quantify the effect sizes the study could detect, we performed a simulation-based power analysis on the primary analysis set (n = 48; 80% power, two-sided α = 0.05). For alpha diversity, genotype-associated shifts of increasing magnitude were added to Shannon values and re-tested with the cage-aware mixed model. For community structure, coherent genotype-associated shifts were injected into the CLR-transformed profiles and re-tested by cage-blocked PERMANOVA. For per-genus differential abundance, the minimum detectable CLR difference was derived from the median per-genus residual variance under Benjamini–Hochberg control across all tested genera.

## Results

### Cohort design and sequencing metrics

We profiled fecal metagenomes from 50 male mice (*Lamp2*KO, n = 29; WT, n = 21) sampled at 3, 6, 9, and 12 months and housed either in single-genotype cages or mixed-genotype cohousing (Supplementary Tables S1–S2 in S1 File). Each sample represents an individual mouse (including in cohoused cages), enabling genotype-resolved comparisons while accounting for cage structure and sequencing batch in all inferential analyses (Methods). Two *Lamp2*KO libraries did not meet the prespecified sequencing-depth threshold (<3 × 10^5^ classified genus-level reads) and were excluded from primary inference. Both excluded samples were *Lamp2*KO mice from single-genotype cages sequenced in batch 3 (run 35309; classified genus-level reads: 9.7 × 10^4^ and 1.6 × 10^5^). The primary analysis set therefore comprised 48 samples (*Lamp2*KO, n = 27; WT, n = 21) from 27 cages, including 12 mixed-genotype cages in which both genotypes were sampled (Supplementary Table S2 in S1 File). Sequencing and classification metrics derived from One Codex are summarized in Table 1, with per-sample metadata, depth, batch, and cage identifiers provided in Supplementary Table S3 in S1 File and depth summaries in Supplementary Table S4 in S1 File. Across all 50 samples, the mean proportion of reads mapped to microbial references was 73.2% ± 8.1% and did not differ by genotype (WT: 72.0% ± 8.4%; *Lamp2*KO: 74.1% ± 7.9%; Welch’s t-test, p = 0.38). The host/non-specific read fraction was higher in *Lamp2*KO than WT (8.5% ± 6.8% vs 4.4% ± 2.6%; p = 0.005). This signal reflected bona fide *Mus musculus* reads (mouse-specific fraction 7.5% vs 3.5% of total reads; p = 0.004) and remained significant after accounting for cage (p = 0.008; Supplementary Fig S3 in S1 File). The host read fraction was included as a covariate in all downstream community analyses, which used compositional approaches as described in Methods; its potential biological interpretation is considered in the Discussion.

**Table 1:** Sequencing and read-classification summary for fecal metagenomic libraries (One Codex).

Metric	Overall (n = 50) Mean ± SD (Range)	WT (n = 21) Mean ± SD	*Lamp2*KO (n = 29) Mean ± SD	p-value (Welch’s t-test)
Mapped reads (%)	73.2 ± 8.1 (55–86)	72.0 ± 8.4	74.1 ± 7.9	0.38
Specific microbial reads (%)	66.3 ± 10.1 (35–83)	67.5 ± 9.1	65.5 ± 10.9	0.48
Non-specific/host reads (%)	6.8 ± 5.8 (0–25)	4.4 ± 2.6	8.5 ± 6.8	**0.005**
Unclassified reads (%)	26.8 ± 8.1 (14–45)	28.0 ± 8.4	25.9 ± 7.9	0.38

Footnote: Values are mean ± SD of percent of total reads; the overall range is shown in parentheses. “Mapped” denotes reads assigned to any reference by the One Codex classifier; “Specific microbial” denotes reads uniquely assigned to microbial taxa; “Non-specific/host” denotes reads assigned to host genomes and/or non-unique classifications; “Unclassified” denotes reads not assigned to any reference (Mapped + Unclassified = 100%, subject to rounding). This table summarizes all sequenced libraries (n = 50; WT n = 21; *Lamp2*KO n = 29), including the two low-depth libraries excluded from the primary inferential analyses. P-values are from two-sided Welch’s t-tests comparing WT vs *Lamp2*KO and are provided for descriptive comparison (not adjusted for multiple comparisons). Per-sample metrics, sequencing batch, and cage identifiers are provided in Supplementary Table S3 in S1 File.

### Genus-level alpha diversity is similar between genotypes

**Fig 1:**
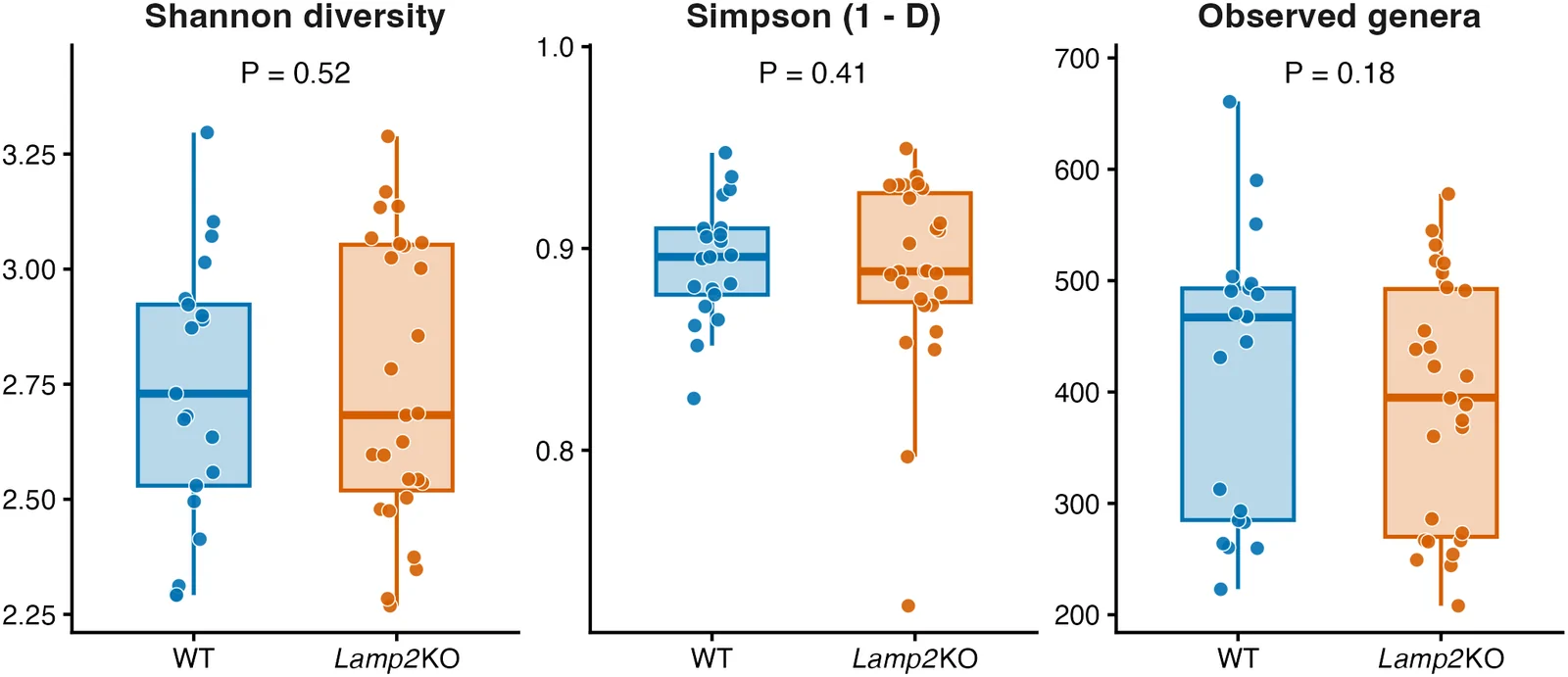
Genus-level alpha diversity is similar between genotypes. Shannon diversity, Simpson evenness (1 − D), and observed genus richness in the primary analysis set (n = 48; WT n = 21, *Lamp2*KO n = 27), computed from prevalence-filtered genus tables. Boxes show median and interquartile range; whiskers extend to 1.5 × IQR; each point is one fecal sample. P-values are for the genotype term from cage-aware linear mixed-effects models (Supplementary Table S8 in S1 File).

In cage-aware mixed-effects models adjusting for housing condition, age group, sequencing batch, and sequencing depth, genotype was not associated with Shannon diversity, Simpson evenness, or observed genus richness (Table 2, Fig 1). Summary statistics were comparable between WT and *Lamp2*KO mice (Shannon: 2.74 ± 0.29 vs 2.75 ± 0.31; Simpson: 0.89 ± 0.03 vs 0.89 ± 0.05; observed genera: 416 ± 126 vs 391 ± 111). Per-sample alpha diversity values are provided in Supplementary Table S5 in S1 File. Across genotypes, alpha-diversity metrics were broadly stable across age groups, and cohousing showed only modest descriptive differences in diversity and richness (Table 2). Together, these data indicate that we did not detect genotype-associated differences in within-sample genus diversity under standard SPF housing, although the available sample size provides limited power to detect small effects.

**Table 2:** Genus-level alpha diversity by genotype, age, and housing configuration (primary analysis set).

Comparison	Group	n	Shannon (mean ± SD, range)	Simpson (1 − D), mean ± SD	Observed genus richness, mean ± SD
**Genotype**	WT	21	2.74 ± 0.29 (2.29–3.30)	0.89 ± 0.03	416 ± 126
	*Lamp2*KO	27	2.75 ± 0.31 (2.27–3.29)	0.89 ± 0.05	391 ± 111
**Age (**WT)	3 months	6	2.63 ± 0.29 (2.29–3.01)	0.88 ± 0.04	438 ± 167
	6 months	8	2.73 ± 0.35 (2.29–3.30)	0.89 ± 0.03	403 ± 132
	9 months	4	2.89 ± 0.18 (2.67–3.10)	0.91 ± 0.02	422 ± 108
	12 months	3	2.77 ± 0.21 (2.53–2.90)	0.90 ± 0.02	402 ± 95
**Age (***Lamp2*KO)	3 months	6	2.59 ± 0.23 (2.35–3.02)	0.88 ± 0.03	331 ± 110
	6 months	12	2.87 ± 0.35 (2.28–3.29)	0.91 ± 0.03	403 ± 128
	9 months	5	2.81 ± 0.20 (2.62–3.06)	0.89 ± 0.02	429 ± 59
	12 months	4	2.53 ± 0.22 (2.27–2.78)	0.83 ± 0.08	395 ± 112
**Housing (**WT)	Cohoused	14	2.77 ± 0.31 (2.29–3.30)	0.89 ± 0.03	444 ± 124
	WT-only	7	2.68 ± 0.25 (2.31–3.07)	0.89 ± 0.02	361 ± 120
**Housing (***Lamp2*KO)	Cohoused	17	2.73 ± 0.30 (2.27–3.17)	0.88 ± 0.05	397 ± 106
	KO-only	10	2.77 ± 0.33 (2.28–3.29)	0.90 ± 0.03	381 ± 125

**Footnote: Values are mean** ± SD**; Shannon ranges are shown in parentheses. Analyses are restricted to samples in the primary analysis set that met the prespecified sequencing-depth threshold (**≥**3** × **10**^5^ **classified genus-level reads; n** = **48 total:** WT **n** = **21,** *Lamp2*KO **n** = **27). Diversity metrics were computed at the genus level using prevalence-filtered count tables (genera present in** ≥**10% of samples in the primary analysis set). Simpson values correspond to 1** − **D (higher values indicate greater evenness). Observed genus richness denotes the number of genera with non-zero counts per sample after prevalence filtering. Housing categories: cohoused** = **mixed-genotype cages;** WT**-only and** KO**-only** = **single-genotype cages. This table is descriptive; statistical inference is reported in the Results using linear mixed-effects models with cage as a random intercept and fixed effects for genotype, housing, age group, sequencing batch, and log10 sequencing depth.**

### Genus-level community composition does not differ by genotype

**Fig 2:**
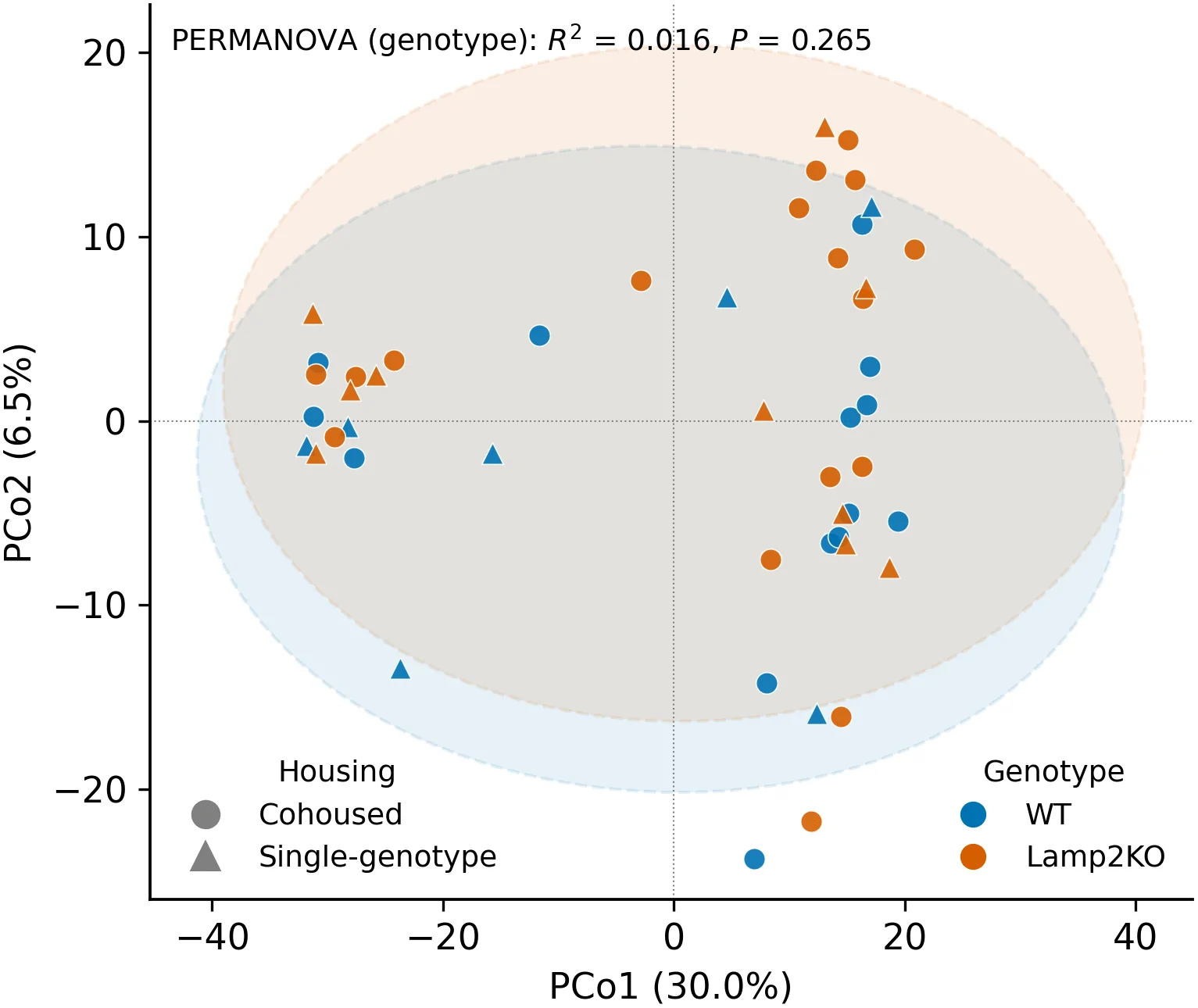
Genus-level community composition does not differ by genotype. Principal coordinate analysis (PCoA) of genus-level Aitchison distances (Euclidean distance on centered-log-ratio–transformed abundances; pseudocount = 0.5) in the primary analysis set (n = 48 samples from 27 cages). Each point represents one fecal sample from an individual mouse; colour denotes genotype (WT, *Lamp2*KO) and shape denotes housing configuration (cohoused vs single-genotype cages). Dashed ellipses are 95% normal-data ellipses for each genotype. Axes show the percent variance explained (PCo1 = 30.0%, PCo2 = 6.5%). Community composition did not differ by genotype in cage-blocked PERMANOVA adjusting for housing configuration, age group, and sequencing batch (genotype: R² = 0.016, P = 0.265; Table 3).

We evaluated beta diversity in the primary analysis set (n = 48) using genus-level profiles and cage-blocked permutation testing. Aitchison distance (Euclidean distance on CLR-transformed abundances) was the primary metric; Bray–Curtis distance on relative abundances was used as a secondary comparison. In cage-blocked PERMANOVA models adjusting for housing condition, age group, and sequencing batch, genotype was not associated with overall community composition (Aitchison: R^2^ = 0.016, p = 0.265; Bray–Curtis: R^2^ = 0.015, p = 0.136; Table 3). No covariate reached statistical significance under either distance metric (all p > 0.1). Both genotypes were represented across all five sequencing batches, so genotype was not confounded with batch; batch was modeled as a covariate and, although it was the largest non-residual term, it was not significant (Table 3). PCoA of Aitchison distances showed substantial overlap across genotype and housing groups (Fig 2); the first two axes explained 30.0% (PC1) and 6.5% (PC2) of variance. Dispersion did not differ by genotype for either distance metric (PERMDISP p = 0.493 for Aitchison; p = 0.482 for Bray–Curtis). Dispersion by housing was similar under Aitchison distance (p = 0.369) but differed under Bray–Curtis (p = 0.020).

**Table 3:** Cage-blocked PERMANOVA of genus-level beta diversity in the primary analysis set.

Term	Aitchison R^2^	Aitchison p	Bray–Curtis R^2^	Bray–Curtis p
Genotype	0.0156	0.265	0.0146	0.136
Housing	0.0500	0.730	0.0489	0.347
Age group	0.1074	0.149	0.1050	0.757
Batch	0.0997	0.115	0.0720	0.831

Footnote: PERMANOVA was performed using adonis2 (vegan) in the primary analysis set (n = 48 samples from 27 cages), using 999 permutations constrained within cages (strata = cage) and marginal sums of squares (by = “margin”) for the model: distance ~ genotype + housing configuration + age group + sequencing batch. Aitchison distance was computed as Euclidean distance on centered-log-ratio (CLR) transformed genus abundances (pseudocount = 0.5) using the prevalence-filtered genus table; Bray–Curtis distance was computed on relative abundances for comparison. Reported R^2^ values indicate the proportion of variance explained by each term in the marginal model. Homogeneity of dispersion was assessed using betadisper (PERMDISP); dispersion differed by housing under Bray–Curtis distance (p = 0.020) but not under Aitchison distance (p = 0.369), and dispersion did not differ by genotype for either distance metric.

### No genera show FDR-significant differential abundance by genotype

**Fig 3:**
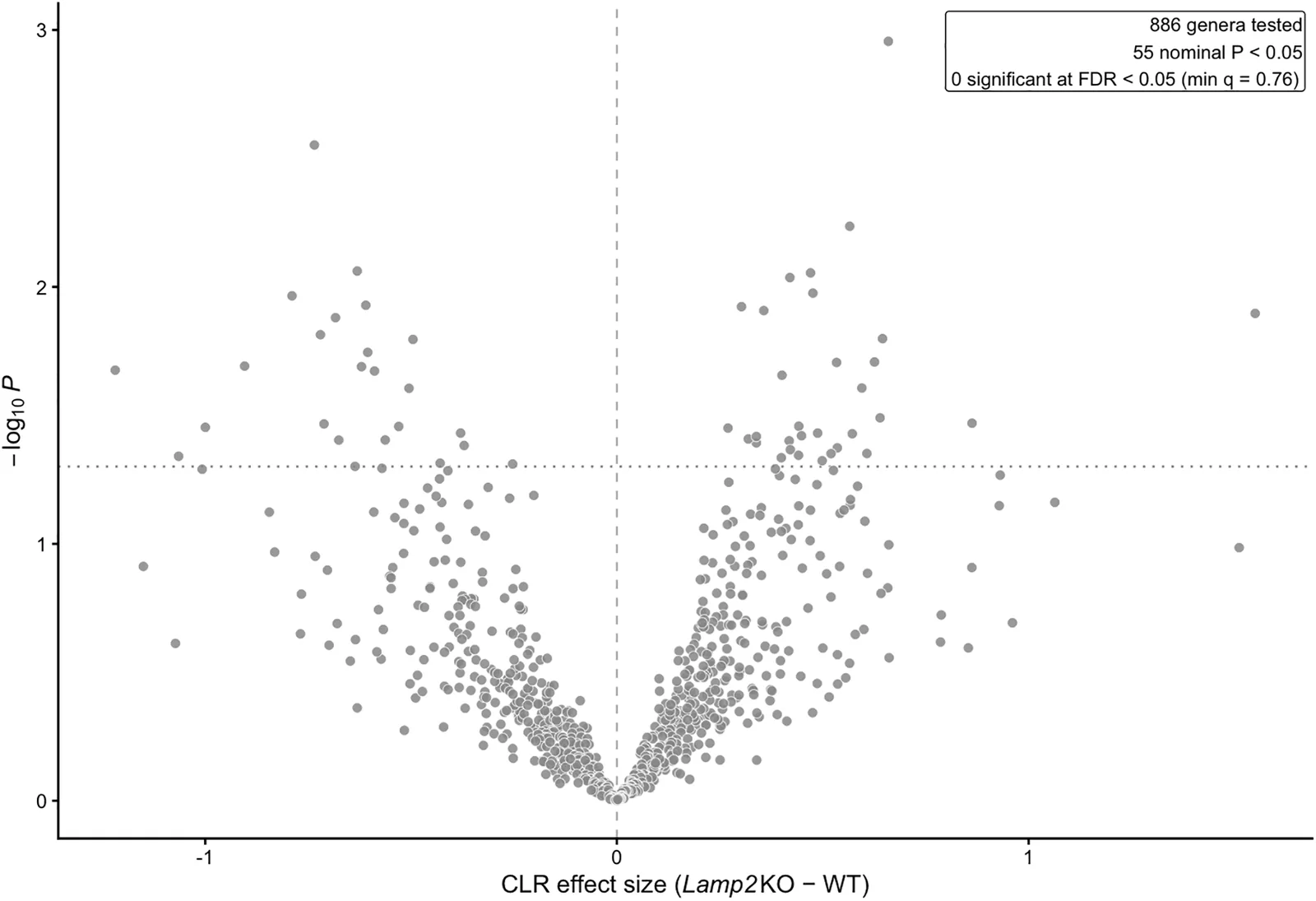
No genera show FDR-significant differential abundance by genotype. Volcano plot of genus-level differential abundance (886 genera, primary analysis set) from cage-aware linear mixed-effects models on centered-log-ratio abundances (genotype contrast *Lamp2*KO − WT, adjusting for housing, age group, sequencing batch, sequencing depth, and host-read fraction). Each point is one genus; the x-axis shows the genotype CLR effect size and the y-axis −log₁₀(P). The dotted line marks nominal P = 0.05. Although 55 genera reached nominal significance, none remained significant after Benjamini–Hochberg correction (minimum q = 0.76).

We tested genus-level differential abundance using CLR-transformed abundances and linear mixed-effects models with cage modeled as a random intercept and fixed effects for genotype, housing condition, age group, sequencing batch, log10 sequencing depth, and host/non-specific read fraction where available (Methods). Across 886 genera, 55 showed nominal genotype associations (p < 0.05), but none remained significant after multiple-testing correction (minimum Benjamini–Hochberg q = 0.76) (Fig 3). Effect sizes were small and were not driven by highly prevalent taxa. These analyses provide no evidence for robust, cage-independent genotype-associated shifts at the genus level in this cohort, while recognizing that the cohort size limits power to detect small or low-prevalence effects. These conclusions were unchanged in a sensitivity analysis including the two excluded low-depth libraries (n = 50; Supplementary Table S8 in S1 File).

### Exploratory within-cage contrasts highlight a small set of low-abundance genera

To further control for shared environment, we performed an exploratory paired-cage analysis restricted to mixed-genotype cages in which both genotypes were sampled (12 paired cages). For each genus, we computed within-cage CLR differences (*Lamp2*KO - WT) and tested whether the median difference across cages differed from zero using the Wilcoxon signed-rank test. Within this paired framework, seven genera met an exploratory threshold of FDR q < 0.10 after restricting to taxa with prevalence ≥20% across paired samples (Supplementary Table S6 in S1 File). *Halomonas*, *Stenotrophomonas*, *Veillonella*, *Pseudomonas*, and *Streptomyces* were lower in *Lamp2*KO, whereas *Thomasclavelia* and *Herbaspirillum* were higher; all were low-abundance taxa (mean relative abundance ≤0.003%).

These paired-cage signals were not supported by the primary cohort-wide mixed-effects models and are therefore considered hypothesis-generating rather than confirmatory. Consistent with a technical origin, six of the seven genera (*Halomonas, Stenotrophomonas, Veillonella, Pseudomonas, Herbaspirillum,* and *Streptomyces*) are recognized environmental or reagent contaminants [14], all were present at trace relative abundance (≤0.003%), and several were relatively enriched in lower-biomass samples (a contamination-consistent pattern); only *Thomasclavelia*, a gut commensal, is not a typical contaminant.

### Taxonomic profiles are broadly similar between *Lamp2KO* and WT mice

**Fig 4:**
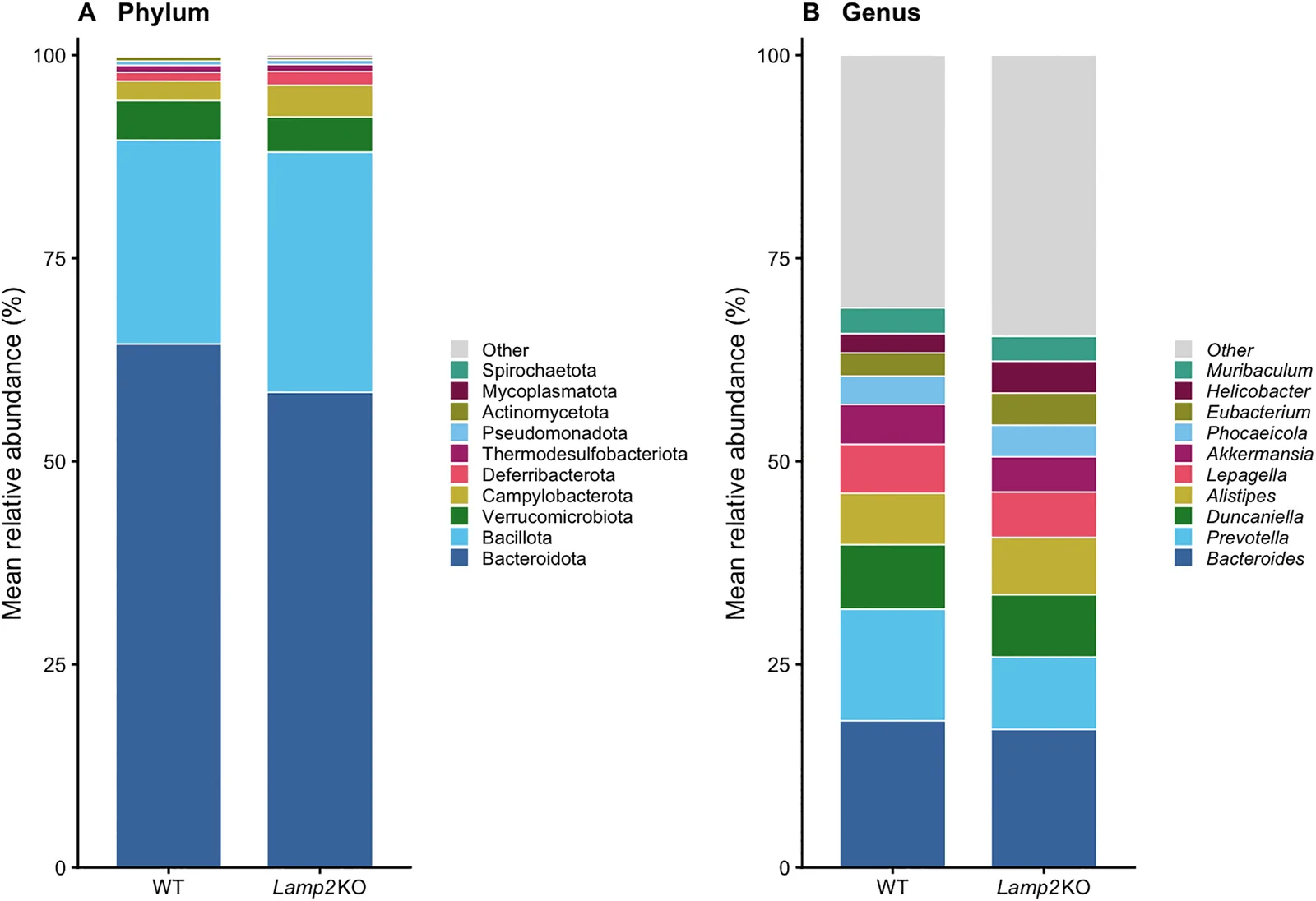
Taxonomic composition is broadly similar between genotypes. Mean relative abundance of the ten most abundant phyla (A) and genera (B) by genotype across all sequenced libraries (n = 50; WT n = 21, *Lamp2*KO n = 29). For each rank, the ten most abundant taxa (ranked by mean relative abundance across samples) are shown; all remaining taxa are pooled as “Other.” Bars represent the mean within each genotype. Genus names are italicized.

Across both genotypes, fecal communities were dominated by Bacteroidota and Bacillota (Fig 4A). The most abundant genera included *Bacteroides*, *Alistipes*, and *Prevotella* (Fig 4B), and frequently detected species included *Akkermansia muciniphila*, *Helicobacter typhlonius*, *Xylanibacter caecicola*, and *Bacteroides acidifaciens* (Supplementary Fig S1 in S1 File). These composition summaries provide context for community structure; inferential testing of genotype effects is reported above using cage-aware mixed-effects and paired-cage analyses.

Formal statistical testing of genotype effects was performed at the genus level using cage-aware mixed-effects models and an exploratory paired-cage analysis (see Results and Supplementary Table S6 in S1 File). Supplementary Figure S1 in S1 File shows per-sample taxonomic composition.

### Inferred functional pathway profiles show minimal genotype-associated differences

Among the 50 most abundant microbial metabolic pathways, overall functional composition was similar between genotypes (**Supplementar**y Fig S2 in S1 File). Cage-blocked PERMANOVA on CLR-transformed pathway abundances (Aitchison distance) did not detect differences attributable to genotype, housing condition, age group, or sequencing batch (all p > 0.1). In pathway-level mixed-effects models, one pathway met an exploratory FDR threshold: dTDP-β-L-rhamnose biosynthesis was lower in *Lamp2*KO mice (CLR estimate −0.104, SE 0.030; q = 0.086; Supplementary Table S7 in S1 File). No pathways met q < 0.05. Together, functional profiling supports minimal genotype-associated differences in dominant pathway abundances in this cohort. Extending the per-pathway analysis to all detected pathways (n = 214) yielded no pathway significant after Benjamini–Hochberg correction (minimum q = 0.23), confirming that focusing on the most abundant pathways did not omit a genotype-associated pathway.

## Discussion

In this study, we used shotgun metagenomics to profile fecal microbial communities in *Lamp2*KO and WT mice across age groups and housing configurations and tested for genotype-associated signals using cage-aware compositional analyses. Across genus-level alpha diversity, beta diversity, and differential abundance, we found no evidence of robust, cage-independent shifts attributable to *Lamp2* loss after multiple-testing correction. Predicted functional profiles were similarly stable [13,16,17].

Within the limits of this cohort’s sample size and design, our data indicate that we did not detect a major effect of *Lamp2* deficiency on fecal microbiome structure under standard SPF husbandry and chow. This conclusion is strengthened by the analytical framework: shared housing is a dominant source of correlation and environmental similarity in murine microbiome studies, and treating cage-mates as independent replicates can inflate apparent genotype effects. By explicitly accounting for cage structure (random effects and cage-blocked permutations), we reduced the likelihood of attributing shared-exposure variation to genotype [3,18,19].

At the taxon level, primary mixed-effects models did not identify any genera with differential abundance that survived FDR correction after adjusting for cage and technical covariates. The exploratory paired-cage analysis (restricted to cages containing both genotypes) identified a small set of genera with consistent within-cage directionality. However, these signals were not supported by the primary mixed-effects models and therefore should be interpreted cautiously and descriptively. Indeed, six of the seven taxa implicated in the paired analysis are recognized environmental or reagent contaminants [14], all were present at trace relative abundance (≤0.003%), and several showed the inverse abundance–depth relationship characteristic of contamination; only *Thomasclavelia* is a typical gut commensal. These signals are therefore most consistent with low-level technical background rather than genotype-driven biology, and independent replication and targeted validation would be required before assigning biological significance to these taxa-level trends [14,15].

Functional profiling converged on the same interpretation. Multivariate testing of abundant pathway profiles did not support genotype-, housing-, age-, or batch-associated shifts in overall functional composition after accounting for cage structure. The isolated association with dTDP-β-L-rhamnose biosynthesis (exploratory q < 0.10) was modest and not part of a broader, coherent pattern. This pathway contributes to bacterial cell-envelope glycan synthesis in many taxa and can relate to surface architecture and host–microbe interactions [20]. Importantly, these pathway profiles represent inferred functional *potential* derived from microbial gene content rather than measured functional activity: shotgun metagenomics quantifies the relative abundance of genes assigned to annotated pathways but does not capture gene expression, enzyme activity, or metabolite production. We did not perform orthogonal biological validation (e.g., metatranscriptomics, targeted metabolomics, or biochemical assays), so the exploratory dTDP-β-L-rhamnose association should be regarded as a hypothesis to be tested directly—for example, by quantifying rhamnose-containing cell-envelope glycans or the corresponding nucleotide-sugar intermediates—rather than as evidence of an altered functional output *in vivo*. As with all reference-based functional inference from metagenomes, pathway-level results additionally reflect relative-abundance patterns conditioned on database annotation and should be interpreted in that context [13,16].

Several interpretations are consistent with the absence of a strong fecal signal. Host systems that shape intestinal homeostasis may buffer lysosome–autophagy perturbations through compensatory programs and redundancy within lysosomal quality-control networks [6]. In parallel, microbial communities can preserve higher-level functional outputs through redundancy across taxa, which can limit detectability of host-genotype effects in coarse functional summaries [21]. In addition, fecal metagenomics captures luminal output and may not reflect mucosa-associated or region-specific communities that interact most directly with epithelial lysosomal stress pathways. Tissue-proximal microbial niches (mucus layer, small intestine, and mucosal surfaces) can show patterns that are not recapitulated in bulk stool, particularly for immune–epithelial phenotypes [1,4,22]. Notably, we observed a higher fecal host-DNA (*Mus musculus*) fraction in *Lamp2*KO mice, which persisted after accounting for cage. Because LAMP2 is required for lysosomal function and autophagy, this may reflect increased intestinal epithelial turnover or shedding in knockout animals; however, technical contributions—such as differences in stool composition or sample handling—cannot be excluded, and the observation should be regarded as hypothesis-generating and warranting direct measurement in future work. The host read fraction was included as a covariate in all differential-abundance models, and our microbial conclusions were unchanged when it was omitted.

Danon disease is a multisystem lysosomal disorder. Beyond cardiomyopathy and skeletal myopathy, additional involvement can include hepatic abnormalities and gastrointestinal disease, as well as retinal disease [7,23]. These systemic features make it biologically reasonable to ask whether *Lamp2* deficiency could influence intestinal physiology and, secondarily, the gut microbial ecosystem. In parallel, an expanding literature supports a “gut–eye” framework in which gut microbial communities and microbial metabolites can modulate retinal inflammation, vascular phenotypes, and degenerative processes in specific experimental contexts, including age-related macular degeneration and immune-mediated uveitis models [24–26]. Preclinical studies have also reported that altering microbial status (e.g., germ-free conditions or microbiome manipulation) can change choroidal neovascularization and retinal/RPE transcriptional programs, consistent with systemic immune–metabolic signaling from the gut to the outer retina [26].

Against this backdrop, our findings provide an important constraint for interpreting Lamp2-associated retinal phenotypes in mice: under standard SPF conditions, *Lamp2*KO mice did not show a robust, cage-independent fecal dysbiosis or broad pathway-level remodeling detectable by shotgun metagenomics. These data therefore suggest that large baseline shifts in bulk fecal microbiome composition are unlikely to be a primary driver of the murine *Lamp2*KO phenotype. At the same time, they do not exclude microbiome contributions that are localized to mucosal compartments, mediated by microbial metabolites without major taxonomic shifts, or revealed only under defined perturbations (dietary challenges, inflammation, antibiotic exposure, or barrier stress). Finally, these mouse results should not be extrapolated to humans; Danon patients have heterogeneous exposures, medications, diets, and disease states that can influence gut ecology in ways not modeled here.

Beyond the *Lamp2* context, this study highlights several broadly applicable points for murine microbiome research. First, cage structure should be treated as a core design feature and explicitly handled in inference, because it defines the shared exposure environment and the effective correlation structure of the data [3,18]. Second, conservative conclusions benefit from convergent analyses that match the statistical properties of microbiome data (compositionality, sparsity, and high dimensionality), coupled with transparent reporting of filtering thresholds and sensitivity analyses [17]. Third, nominal signals, especially in low-abundance taxa, should be interpreted conservatively unless supported across complementary models and robust to multiple-testing correction and plausible technical artifacts [14,15]. In aggregate, these practices improve reproducibility and help distinguish true host-intrinsic effects from shared-environment variation.

This study has strengths that support the credibility of its negative primary findings. Foremost is the use of shotgun metagenomics, which provides higher taxonomic resolution (to the species level here) and direct functional-potential profiling, increasing sensitivity to both compositional and metabolic genotype effects; that a method of this resolution detected no robust, cage-independent shift strengthens the inference that *Lamp2* loss does not substantially remodel the bulk fecal community. Additional strengths include age-stratified sampling, mixed housing configurations that included mixed-genotype cohousing, and cage-aware inferential modeling. Environmental variation, a dominant driver of murine microbiome composition, was minimized by design: control and knockout animals were generated as littermates from heterozygous dams, housed in a single SPF facility on a common diet and water source provided ad libitum, sampled at a standardized time of day, and analyzed with cage modeled as a random effect. However, residual unmeasured environmental variation cannot be fully excluded.

Limitations define the boundaries of interpretation. The study is feces-based and performed under baseline SPF husbandry and chow; it does not address mucosal or region-specific communities, small-intestinal niches, or stress-dependent microbiome phenotypes. Shotgun profiling remains a relative-abundance measurement and depends on reference-based inference for taxonomy and pathway annotation [16,17]. Critically, all functional results reflect predicted pathway potential inferred from metagenomic gene content and require direct biological validation before mechanistic interpretation.

Because *Lamp2* is X-linked and the study was, by design, restricted to hemizygous males to obtain a uniform null genotype, our findings may not generalize to heterozygous female carriers, and sex-specific or sex-by-genotype microbiome effects could not be evaluated. Fecal sampling was also cross-sectional: although the cohort spanned four ages (3–12 months), each animal contributed a single sample rather than repeated longitudinal measurements. We therefore captured an age-stratified snapshot of community structure but could not assess within-animal temporal stability, individual trajectories, or age-by-genotype dynamics; a longitudinal design with repeated sampling of the same animals would be required to detect transient or time-dependent genotype effects.

Finally, effective replication is constrained by cage structure (cage accounted for approximately half the variance in alpha diversity; intraclass correlation ≈ 0.48), which limits statistical power. By simulation, the study had approximately 80% power to detect a between-genotype difference of ≥ 0.20 in Shannon diversity (Cohen’s d ≈ 0.7; observed difference 0.01) and a coherent community-level shift exceeding the variance expected for a random grouping (observed genotype R² = 0.016, below the chance expectation of ≈ 0.022); for per-genus differential abundance, a genus of typical variance would have required a ≈ 3.5-fold difference (centered-log-ratio ≥ 1.25) to be detected after correction across 886 genera. The study was therefore positioned to detect moderate-to-large genotype effects but cannot exclude smaller or low-prevalence effects, and the negative findings should be interpreted as no detectable effect above these magnitudes.

## Conclusions

In summary, this work establishes a conservative baseline: under standard SPF conditions, we did not detect robust, reproducible shifts in fecal microbiome composition or dominant inferred functional pathway profiles attributable to *Lamp2* deficiency when cage structure and compositionality are handled explicitly; the cohort size and single-time-point, feces-based design constrain the effect magnitudes that can be excluded. If *Lamp2*-dependent murine microbiome effects exist, they are likely modest at baseline, localized to tissue-proximal compartments, mediated through metabolite-level signaling, or revealed under defined perturbations. These results provide a useful reference point for future studies of lysosomal/autophagy disorders and for interpreting systemic phenotypes, including Danon-associated cardiomyopathy and retinopathy, in the context of host–microbe interactions.

## Supporting information

S1 File Supporting tables and figures. Tables S1–S8 and Figures S1–S3, as cited in the main text.
(DOCX)
