# Supplementary material for: Shotgun metagenomic profiling of the fecal microbiome in Lamp2 knockout mice reveals limited genotype-associated differences under standard housing
Source: PLoS One. 2026 Sep 18;21(9):e0357009. doi: 10.1371/journal.pone.0357009 (PMC13588336; doi:10.1371/journal.pone.0357009)
Supplement: Supplementary file 1 [file pone.0357009.s001.docx]

**Supplement**

**Supplementary Table S1. Sample counts by genotype, age, and housing configuration in the primary analysis set (n = 48)**

| **Age group** | **Total n** | ***Lamp2*KO n** | **WT n** | ***Lamp2*KO housing breakdown** | **WT housing breakdown** |
| --- | --- | --- | --- | --- | --- |
| 3 months | 12 | 6 | 6 | Cohoused: 6 | Cohoused: 6 |
| 6 months | 20 | 12 | 8 | KO-only: 6; Cohoused: 6 | WT-only: 4; Cohoused: 4 |
| 9 months | 9 | 5 | 4 | KO-only: 2; Cohoused: 3 | WT-only: 1; Cohoused: 3 |
| 12 months | 7 | 4 | 3 | KO-only: 2; Cohoused: 2 | WT-only: 2; Cohoused: 1 |
| **Total** | **48** | **27** | **21** | KO-only: 10; Cohoused: 17 | WT-only: 7; Cohoused: 14 |

Footnotes: All samples were collected from male mice maintained on standard chow under specific pathogen–free conditions. Housing categories: KO-only = *Lamp2*KO-only cages; WT-only = WT-only cages; cohoused = mixed-genotype *Lamp2*KO–WT cages. Counts reflect the primary analysis set after exclusion of two low-depth libraries (<3 × 10^5^ classified genus-level reads). Cage structure was explicitly accounted for in all inferential analyses (random effects or permutation strata), as described in the Methods.

**Supplementary Table S2. Cage-level sampling structure and genotype composition in the primary analysis set**

Each row represents a cage. “Total samples” indicates the number of fecal samples collected from that cage across all sampled ages; “Age groups sampled” indicates the ages represented for that cage. This table documents the shared-environment structure used for cage-aware inference and is not itself a unit-of-inference table.

| **Cage ID** | **Cage composition** | **Genotypes sampled** | **Total samples** | **Age groups sampled (months)** |
| --- | --- | --- | --- | --- |
| 1090 | KO-only | *Lamp2*KO | 1 | 12 |
| 1162 | WT-only | WT | 1 | 12 |
| 1184 | WT-only | WT | 1 | 12 |
| 1192 | KO-only | *Lamp2*KO | 1 | 12 |
| 1193 | Mixed-genotype | *Lamp2*KO + WT | 3 | 12 |
| 1238 | KO-only | *Lamp2*KO | 1 | 9 |
| 1248 | Mixed-genotype | *Lamp2*KO + WT | 2 | 9 |
| 1266 | KO-only | *Lamp2*KO | 1 | 6 |
| 1277 | WT-only | WT | 1 | 6 |
| 1290 | WT-only | WT | 1 | 6 |
| 1298 | KO-only | *Lamp2*KO | 1 | 6 |
| 1309 | KO-only | *Lamp2*KO | 2 | 6, 9 |
| 1320 | Mixed-genotype | *Lamp2*KO + WT | 5 | 6, 9 |
| 1332 | Mixed-genotype | *Lamp2*KO + WT | 5 | 6, 9 |
| 1334 | WT-only | WT | 1 | 9 |
| 1342 | WT-only | WT | 1 | 6 |
| 1346 | Mixed-genotype | *Lamp2*KO + WT | 2 | 6 |
| 1385 | Mixed-genotype | *Lamp2*KO + WT | 2 | 6 |
| 1407 | KO-only | *Lamp2*KO | 1 | 6 |
| 1415 | WT-only | WT | 1 | 6 |
| 1416 | KO-only | *Lamp2*KO | 2 | 6 |
| 1507 | Mixed-genotype | *Lamp2*KO + WT | 2 | 3 |
| 1516 | Mixed-genotype | *Lamp2*KO + WT | 2 | 3 |
| 1525 | Mixed-genotype | *Lamp2*KO + WT | 2 | 3 |
| 1535 | Mixed-genotype | *Lamp2*KO + WT | 2 | 3 |
| 1536 | Mixed-genotype | *Lamp2*KO + WT | 2 | 3 |
| 1538 | Mixed-genotype | *Lamp2*KO + WT | 2 | 3 |

Footnotes: All samples were collected from male mice maintained on standard chow under specific pathogen–free conditions. Cage composition: KO-only = *Lamp2*KO -only cages; WT-only = WT-only cages; mixed-genotype = cages containing both *Lamp2*KO and WT mice. Some cages were sampled at more than one age group; statistical models accounted for cage as a random effect or permutation stratum. Two *Lamp2*KO samples with <3 × 10^5^ classified genus-level reads were excluded from primary inferential analyses; all cages/samples remaining constitute the primary analysis set. Cage was not treated as the unit of inference.

**Supplementary Table S3. Per-sample metadata and sequencing/classification metrics reported by One Codex (descriptive; all libraries)**

Per-sample metadata and One Codex summary metrics are reported for all sequenced libraries (n = 50), including the two low-depth *Lamp2*KO libraries excluded from primary inference. Metrics include total reads and mapped reads (in millions), mapped percent, percent assigned to specific microbial classifications, and percent unclassified.

A. ***Lamp2*KO samples (n=29)**

| **Sample ID** | **Age (mos)** | **Housing** | **Total reads (M)** | **Mapped reads (M)** | **Mapped (%)** | **Specific Microbial (%)** | **Unclassified (%)** | **Seq. Batch** | **Cage** |
| --- | --- | --- | --- | --- | --- | --- | --- | --- | --- |
| MB00008288 | 3 | With WT | 7.4 | 6.2 | 84 | 72 | 16 | 2 | 25 |
| MB00008593 | 3 | With WT | 7.5 | 5.6 | 74 | 69 | 26 | 4 | 27 |
| MB00008620 | 3 | With WT | 9.4 | 7.4 | 78 | 72 | 22 | 4 | 26 |
| MB00009015 | 3 | With WT | 13.0 | 9.4 | 73 | 50 | 27 | 4 | 28 |
| MB00009097 | 3 | With WT | 3.6 | 3.0 | 82 | 81 | 18 | 2 | 23 |
| MB00009111 | 3 | With WT | 4.8 | 4.0 | 83 | 79 | 17 | 2 | 24 |
| MB00008905 | 6 | With WT | 4.3 | 3.1 | 71 | 68 | 29 | 2 | 13 |
| MB00009083 | 6 | With WT | 6.6 | 4.1 | 62 | 53 | 38 | 2 | 14 |
| MB00009183 | 6 | With WT | 8.4 | 6.6 | 79 | 76 | 21 | 1 | 17 |
| MB00009192 | 6 | With WT | 6.5 | 5.0 | 77 | 52 | 23 | 1 | 13 |
| MB00009362 | 6 | With WT | 4.1 | 2.8 | 67 | 62 | 33 | 3 | 18 |
| MB00009388 | 6 | With WT | 13.4 | 8.3 | 62 | 54 | 38 | 1 | 14 |
| MB00009138 | 6 | KO-only | 9.5 | 8.1 | 86 | 78 | 14 | 1 | 11 |
| MB00009193 | 6 | KO-only | 0.3 | 0.2 | 74 | 53 | 26 | 3 | 19 |
| MB00009198 | 6 | KO-only | 10.0 | 6.7 | 67 | 64 | 33 | 1 | 12 |
| MB00009212 | 6 | KO-only | 6.2 | 5.2 | 83 | 76 | 17 | 3 | 21 |
| MB00009372 | 6 | KO-only | 0.8 | 0.4 | 57 | 35 | 43 | 3 | 22 |
| MB00009395 | 6 | KO-only | 7.9 | 6.6 | 84 | 80 | 16 | 3 | 21 |
| MB00009403 | 6 | KO-only | 5.6 | 3.2 | 58 | 54 | 42 | 3 | 19 |
| MB00009404 | 6 | KO-only | 15.5 | 12.3 | 79 | 63 | 21 | 1 | 8 |
| MB00008991 | 9 | With WT | 5.9 | 4.1 | 70 | 61 | 30 | 5 | 13 |
| MB00009208 | 9 | With WT | 5.3 | 4.1 | 77 | 62 | 23 | 3 | 7 |
| MB00009408 | 9 | With WT | 8.3 | 6.1 | 73 | 64 | 27 | 5 | 14 |
| MB00009220 | 9 | KO-only | 8.1 | 6.3 | 77 | 73 | 23 | 5 | 12 |
| MB00009394 | 9 | KO-only | 3.5 | 2.2 | 65 | 62 | 35 | 3 | 6 |
| MB00009376 | 12 | With WT | 7.0 | 5.4 | 77 | 74 | 23 | 5 | 5 |
| MB00009384 | 12 | With WT | 4.9 | 3.8 | 77 | 71 | 23 | 5 | 5 |
| MB00009021 | 12 | KO-only | 15.5 | 12.2 | 79 | 73 | 21 | 4 | 1 |
| MB00009415 | 12 | KO-only | 7.4 | 5.5 | 74 | 68 | 26 | 5 | 4 |

B. **WT samples (n=21)**

| **Sample ID** | **Age (mos)** | **Housing** | **Total reads (M)** | **Mapped reads (M)** | **Mapped (%)** | **Specific Microbial (%)** | **Unclassified (%)** | **Seq. Batch** | **Cage** |
| --- | --- | --- | --- | --- | --- | --- | --- | --- | --- |
| MB00008297 | 3 | With KO | 21.8 | 13.1 | 60 | 55 | 40 | 4 | 28 |
| MB00008608 | 3 | With KO | 25.2 | 18.1 | 72 | 69 | 28 | 4 | 27 |
| MB00008909 | 3 | With KO | 6.8 | 5.7 | 84 | 80 | 16 | 2 | 24 |
| MB00009023 | 3 | With KO | 10.3 | 7.1 | 69 | 62 | 31 | 4 | 26 |
| MB00009101 | 3 | With KO | 7.6 | 6.2 | 82 | 78 | 18 | 2 | 25 |
| MB00009120 | 3 | With KO | 7.7 | 6.3 | 82 | 81 | 18 | 2 | 23 |
| MB00008287 | 6 | With KO | 9.0 | 6.4 | 71 | 65 | 29 | 2 | 13 |
| MB00008890 | 6 | With KO | 7.0 | 4.9 | 70 | 60 | 30 | 2 | 14 |
| MB00009364 | 6 | With KO | 9.5 | 8.6 | 86 | 83 | 14 | 1 | 17 |
| MB00009389 | 6 | With KO | 6.1 | 3.7 | 61 | 59 | 39 | 3 | 18 |
| MB00008617 | 6 | WT-only | 20.8 | 15.6 | 75 | 74 | 25 | 4 | 20 |
| MB00009176 | 6 | WT-only | 12.6 | 8.3 | 66 | 62 | 34 | 1 | 16 |
| MB00009182 | 6 | WT-only | 9.7 | 7.9 | 82 | 76 | 18 | 1 | 9 |
| MB00009380 | 6 | WT-only | 7.7 | 5.6 | 73 | 70 | 27 | 1 | 10 |
| MB00009253 | 9 | With KO | 7.5 | 5.4 | 72 | 64 | 28 | 5 | 14 |
| MB00009374 | 9 | With KO | 5.9 | 3.9 | 66 | 61 | 34 | 3 | 7 |
| MB00009400 | 9 | With KO | 7.2 | 4.0 | 55 | 48 | 45 | 5 | 13 |
| MB00008603 | 9 | WT-only | 3.0 | 2.0 | 65 | 64 | 35 | 5 | 15 |
| MB00009387 | 12 | With KO | 5.5 | 3.9 | 72 | 66 | 28 | 5 | 5 |
| MB00009223 | 12 | WT-only | 4.3 | 3.4 | 80 | 74 | 20 | 5 | 3 |
| MB00009367 | 12 | WT-only | 5.8 | 4.0 | 69 | 67 | 31 | 5 | 2 |

Notes: Total reads and mapped reads are reported in millions (M). “Mapped (%)” denotes reads assigned to any reference by the One Codex classifier; “Specific microbial (%)” denotes reads uniquely assigned to microbial taxa; “Unclassified (%)” denotes reads not assigned to any reference. Housing categories: cohoused = mixed-genotype *Lamp2*KO–WT cages; KO-only and WT-only = single-genotype cages. Sequencing batch identifiers correspond to sequencing runs: 1 = 35280; 2 = 35292; 3 = 35309; 4 = 35354; 5 = 35371. Shared cage identifiers denote cohoused (mixed-genotype) cages.

**Supplementary Table S4. Sequencing depth (classified genus-level reads) and prespecified exclusion threshold (descriptive)**

| **Group** | **n_total** | **n_included_primary** | **n_excluded_low_depth** | **Classified genus reads, median [IQR]** |
| --- | --- | --- | --- | --- |
| Overall | 50 | 48 | 2 | 2,878,892 [1,895,746, 3,534,026] |
| WT | 21 | 21 | 0 | 3,143,490 [2,043,871, 3,545,275] |
| *Lamp2*KO | 29 | 27 | 2 | 2,610,469 [1,751,545, 3,437,390] |

Footnote: Sequencing depth is defined as the number of classified genus-level reads (sum across genera) used for downstream genus-level analyses. Two *Lamp2*KO libraries with <3 × 10^5^ classified genus-level reads were excluded from primary inferential analyses.

**Supplementary Table S5. Per-sample genus-level alpha diversity metrics (descriptive)**

| **Sample ID** | **Genotype** | **Age (months)** | **Housing** | **Shannon** | **Simpson (1−D)** | **Observed genera** | **Included in primary analysis** |
| --- | --- | --- | --- | --- | --- | --- | --- |
| MB00008297 | WT | 3 | With KO | 2.936 | 0.910 | 661 | Yes |
| MB00008608 | WT | 3 | With KO | 2.635 | 0.882 | 590 | Yes |
| MB00008909 | WT | 3 | With KO | 2.294 | 0.826 | 313 | Yes |
| MB00009023 | WT | 3 | With KO | 3.015 | 0.927 | 493 | Yes |
| MB00009101 | WT | 3 | With KO | 2.413 | 0.865 | 283 | Yes |
| MB00009120 | WT | 3 | With KO | 2.495 | 0.880 | 285 | Yes |
| MB00008287 | WT | 6 | With KO | 2.681 | 0.871 | 491 | Yes |
| MB00008617 | WT | 6 | WT-only | 2.730 | 0.881 | 431 | Yes |
| MB00008890 | WT | 6 | With KO | 2.923 | 0.906 | 504 | Yes |
| MB00009176 | WT | 6 | WT-only | 3.072 | 0.929 | 551 | Yes |
| MB00009182 | WT | 6 | WT-only | 2.312 | 0.852 | 264 | Yes |
| MB00009364 | WT | 6 | With KO | 2.292 | 0.862 | 223 | Yes |
| MB00009380 | WT | 6 | WT-only | 2.559 | 0.897 | 260 | Yes |
| MB00009389 | WT | 6 | With KO | 3.297 | 0.947 | 497 | Yes |
| MB00008603 | WT | 9 | WT-only | 2.673 | 0.895 | 260 | Yes |
| MB00009253 | WT | 9 | With KO | 2.896 | 0.910 | 467 | Yes |
| MB00009374 | WT | 9 | With KO | 2.873 | 0.896 | 471 | Yes |
| MB00009400 | WT | 9 | With KO | 3.103 | 0.936 | 488 | Yes |
| MB00009223 | WT | 12 | WT-only | 2.530 | 0.877 | 293 | Yes |
| MB00009367 | WT | 12 | WT-only | 2.890 | 0.904 | 468 | Yes |
| MB00009387 | WT | 12 | With KO | 2.899 | 0.907 | 445 | Yes |
| MB00008288 | *Lamp2*KO | 3 | With WT | 2.348 | 0.850 | 267 | Yes |
| MB00008593 | *Lamp2*KO | 3 | With WT | 2.479 | 0.889 | 267 | Yes |
| MB00008620 | *Lamp2*KO | 3 | With WT | 2.535 | 0.878 | 414 | Yes |
| MB00009015 | *Lamp2*KO | 3 | With WT | 3.025 | 0.925 | 518 | Yes |
| MB00009097 | *Lamp2*KO | 3 | With WT | 2.597 | 0.887 | 249 | Yes |
| MB00009111 | *Lamp2*KO | 3 | With WT | 2.543 | 0.859 | 273 | Yes |
| MB00008905 | *Lamp2*KO | 6 | With WT | 3.168 | 0.931 | 440 | Yes |
| MB00009083 | *Lamp2*KO | 6 | With WT | 3.137 | 0.936 | 507 | Yes |
| MB00009138 | *Lamp2*KO | 6 | KO-only | 2.374 | 0.872 | 208 | Yes |
| MB00009183 | *Lamp2*KO | 6 | With WT | 2.503 | 0.889 | 244 | Yes |
| MB00009192 | *Lamp2*KO | 6 | With WT | 2.855 | 0.910 | 368 | Yes |
| MB00009193 | *Lamp2*KO | 6 | KO-only | 2.859 | 0.892 | 156 | **No** |
| MB00009198 | *Lamp2*KO | 6 | KO-only | 3.289 | 0.950 | 545 | Yes |
| MB00009212 | *Lamp2*KO | 6 | KO-only | 2.543 | 0.889 | 254 | Yes |
| MB00009362 | *Lamp2*KO | 6 | With WT | 3.134 | 0.932 | 455 | Yes |
| MB00009372 | *Lamp2*KO | 6 | KO-only | 3.053 | 0.928 | 242 | **No** |
| MB00009388 | *Lamp2*KO | 6 | With WT | 3.051 | 0.930 | 578 | Yes |
| MB00009395 | *Lamp2*KO | 6 | KO-only | 2.284 | 0.853 | 286 | Yes |
| MB00009403 | *Lamp2*KO | 6 | KO-only | 3.067 | 0.931 | 438 | Yes |
| MB00009404 | *Lamp2*KO | 6 | KO-only | 3.055 | 0.932 | 516 | Yes |
| MB00008991 | *Lamp2*KO | 9 | With WT | 3.002 | 0.909 | 491 | Yes |
| MB00009208 | *Lamp2*KO | 9 | With WT | 2.683 | 0.872 | 395 | Yes |
| MB00009220 | *Lamp2*KO | 9 | KO-only | 2.687 | 0.888 | 375 | Yes |
| MB00009394 | *Lamp2*KO | 9 | KO-only | 3.058 | 0.913 | 389 | Yes |
| MB00009408 | *Lamp2*KO | 9 | With WT | 2.625 | 0.875 | 494 | Yes |
| MB00009021 | *Lamp2*KO | 12 | KO-only | 2.783 | 0.902 | 532 | Yes |
| MB00009376 | *Lamp2*KO | 12 | With WT | 2.269 | 0.723 | 423 | Yes |
| MB00009384 | *Lamp2*KO | 12 | With WT | 2.475 | 0.797 | 360 | Yes |
| MB00009415 | *Lamp2*KO | 12 | KO-only | 2.596 | 0.883 | 266 | Yes |

Footnotes: Alpha diversity metrics were computed at the genus level from prevalence-filtered count tables (genera present in ≥10% of samples in the primary analysis set). Simpson values correspond to 1 − D. Observed genera denote the number of genera with non-zero counts per sample after prevalence filtering. “Included in primary analysis” indicates whether the sample met the sequencing-depth threshold (≥3 × 10^5^ classified genus-level reads). Housing categories: cohoused = mixed-genotype cages; KO-only and WT-only = single-genotype cages.

**Supplementary Table S6. Exploratory paired-cage genus-level differences within mixed-genotype cohoused cages**

Genera shown were identified in an exploratory paired-cage analysis restricted to mixed-genotype cages in which both genotypes were sampled (12 paired cages; 24 samples). For each genus, within-cage CLR differences were computed as (*Lamp2*KO − WT) and evaluated across cages using the Wilcoxon signed-rank test. The table reports the mean paired CLR difference, the Wilcoxon p-value, the Benjamini–Hochberg FDR-adjusted q-value, and descriptive mean relative abundance and prevalence across the paired samples. To reduce sparsity-driven artifacts, results are restricted to genera with prevalence ≥20% across paired samples and q < 0.10. These findings are hypothesis-generating; primary inference was based on cohort-wide mixed-effects models accounting for cage and technical covariates.

| **Genus** | **n cages** | **Mean CLR difference (KO − WT)** | **Wilcoxon p** | **FDR q** | **Mean relative abundance (%)** | **Prevalence (%)** |
| --- | --- | --- | --- | --- | --- | --- |
| *Halomonas* | 12 | −0.576 | 0.0010 | 0.048 | 0.00038 | 100 |
| *Stenotrophomonas* | 12 | −0.928 | 0.0015 | 0.065 | 0.00016 | 87 |
| *Veillonella* | 12 | −1.009 | 0.0015 | 0.065 | 0.00065 | 94 |
| *Pseudomonas* | 12 | −0.367 | 0.0024 | 0.090 | 0.0030 | 100 |
| *Thomasclavelia* | 12 | +1.046 | 0.0024 | 0.090 | 0.00077 | 100 |
| *Herbaspirillum* | 12 | +0.737 | 0.0024 | 0.090 | 0.00006 | 48 |
| *Streptomyces* | 12 | −0.693 | 0.0024 | 0.090 | 0.0020 | 100 |

Footnote: CLR values were computed using a pseudocount of 0.5. Six of the seven genera (all except *Thomasclavelia*) are recognized environmental or reagent contaminants (Salter et al. [14]) and are present at trace relative abundance (≤0.003%); none reached significance in the primary cohort-wide models.

**Supplementary Table S7. Mixed-effects differential abundance analysis of the 50 most abundant microbial metabolic pathways (*Lamp2*KO vs WT)**

Pathway-level differential abundance was assessed in the primary analysis set using linear mixed-effects models applied to CLR-transformed pathway abundances (One Codex MetaCyc CPM outputs). Effect estimates represent CLR differences between *Lamp2*KO and WT mice (*Lamp2*KO − WT). Models included cage as a random intercept and adjusted for housing configuration, age group, sequencing batch, log10 sequencing depth, and host fraction where available. P-values were adjusted across pathways using the Benjamini–Hochberg false discovery rate (q value BH-FDR). Singular fit indicates boundary model fits; direction reflects the sign of the CLR estimate.

| **Pathway** | **CLR estimate (KO − WT)** | **SE** | **t value** | **p value** | **Singular fit** | **q value (BH-FDR)** | ***Direction*** |
| --- | --- | --- | --- | --- | --- | --- | --- |
| dTDP-beta-L-rhamnose biosynthesis | -0.1043 | 0.03 | -3.534 | 0.0017 | FALSE | 0.086 | *Lower in Lamp2KO* |
| tRNA charging | -0.077 | 0.034 | -2.256 | 0.0335 | FALSE | 0.733 | *Lower in Lamp2KO* |
| folate transformations II (plants) | -0.084 | 0.042 | -2.017 | 0.0534 | FALSE | 0.733 | *Lower in Lamp2KO* |
| L-lysine biosynthesis VI | 0.0666 | 0.035 | 1.918 | 0.0631 | TRUE | 0.733 | *Higher in Lamp2KO* |
| superpathway of fatty acid biosynthesis initiation (E. coli) | 0.0525 | 0.028 | 1.865 | 0.0781 | FALSE | 0.733 | *Higher in Lamp2KO* |
| coenzyme A biosynthesis I (prokaryotic) | 0.0574 | 0.034 | 1.704 | 0.0971 | TRUE | 0.733 | *Higher in Lamp2KO* |
| L-lysine biosynthesis III | 0.0714 | 0.044 | 1.622 | 0.117 | FALSE | 0.733 | *Higher in Lamp2KO* |
| PWY-4242 | 0.051 | 0.033 | 1.561 | 0.127 | TRUE | 0.733 | *Higher in Lamp2KO* |
| superpathway of coenzyme A biosynthesis I (bacteria) | 0.0409 | 0.027 | 1.535 | 0.137 | FALSE | 0.733 | *Higher in Lamp2KO* |
| L-histidine biosynthesis | 0.063 | 0.042 | 1.505 | 0.147 | FALSE | 0.733 | *Higher in Lamp2KO* |
| adenine and adenosine salvage III | -0.0781 | 0.057 | -1.36 | 0.186 | FALSE | 0.821 | *Lower in Lamp2KO* |
| peptidoglycan biosynthesis III (mycobacteria) | 0.0292 | 0.022 | 1.324 | 0.197 | FALSE | 0.821 | *Higher in Lamp2KO* |
| flavin biosynthesis I (bacteria and plants) | 0.0435 | 0.037 | 1.162 | 0.255 | FALSE | 0.982 | *Higher in Lamp2KO* |
| adenosine ribonucleotides de novo biosynthesis | -0.0469 | 0.045 | -1.052 | 0.301 | FALSE | 0.995 | *Lower in Lamp2KO* |
| superpathway of coenzyme A biosynthesis III (mammals) | 0.0329 | 0.032 | 1.031 | 0.309 | TRUE | 0.995 | *Higher in Lamp2KO* |
| L-isoleucine biosynthesis III | 0.0239 | 0.024 | 0.988 | 0.337 | FALSE | 0.995 | *Higher in Lamp2KO* |
| superpathway of guanosine nucleotides de novo biosynthesis I | 0.0507 | 0.065 | 0.785 | 0.438 | TRUE | 0.995 | *Higher in Lamp2KO* |
| inosine-5'-phosphate biosynthesis I | -0.0325 | 0.047 | -0.697 | 0.49 | TRUE | 0.995 | *Lower in Lamp2KO* |
| superpathway of guanosine nucleotides de novo biosynthesis II | 0.0491 | 0.07 | 0.697 | 0.49 | TRUE | 0.995 | *Higher in Lamp2KO* |
| phosphopantothenate biosynthesis I | 0.0218 | 0.034 | 0.652 | 0.519 | TRUE | 0.995 | *Higher in Lamp2KO* |
| peptidoglycan biosynthesis I (meso-diaminopimelate containing) | -0.0124 | 0.02 | -0.635 | 0.531 | FALSE | 0.995 | *Lower in Lamp2KO* |
| queuosine biosynthesis I (de novo) | -0.029 | 0.048 | -0.61 | 0.546 | TRUE | 0.995 | *Lower in Lamp2KO* |
| UMP biosynthesis I | -0.0188 | 0.031 | -0.597 | 0.556 | FALSE | 0.995 | *Lower in Lamp2KO* |
| superpathway of adenosine nucleotides de novo biosynthesis II | 0.0275 | 0.057 | 0.482 | 0.633 | TRUE | 0.995 | *Higher in Lamp2KO* |
| 5-aminoimidazole ribonucleotide biosynthesis I | -0.017 | 0.037 | -0.465 | 0.645 | TRUE | 0.995 | *Lower in Lamp2KO* |
| glycolysis IV | -0.0232 | 0.05 | -0.464 | 0.646 | FALSE | 0.995 | *Lower in Lamp2KO* |
| CDP-diacylglycerol biosynthesis I | -0.0178 | 0.04 | -0.441 | 0.663 | FALSE | 0.995 | *Lower in Lamp2KO* |
| CDP-diacylglycerol biosynthesis II | -0.0178 | 0.04 | -0.441 | 0.663 | FALSE | 0.995 | *Lower in Lamp2KO* |
| cis-vaccenate biosynthesis | -0.0159 | 0.039 | -0.412 | 0.683 | FALSE | 0.995 | *Lower in Lamp2KO* |
| UDP-N-acetylmuramoyl-pentapeptide biosynthesis I (meso-diaminopimelate containing) | -0.008 | 0.02 | -0.393 | 0.698 | FALSE | 0.995 | *Lower in Lamp2KO* |
| methylerythritol phosphate pathway I | -0.0061 | 0.016 | -0.385 | 0.703 | FALSE | 0.995 | *Lower in Lamp2KO* |
| UDP-N-acetylmuramoyl-pentapeptide biosynthesis II (lysine-containing) | -0.0083 | 0.022 | -0.379 | 0.708 | FALSE | 0.995 | *Lower in Lamp2KO* |
| superpathway of aromatic amino acid biosynthesis | 0.0176 | 0.047 | 0.373 | 0.711 | TRUE | 0.995 | *Higher in Lamp2KO* |
| inosine-5'-phosphate biosynthesis II | -0.0178 | 0.051 | -0.352 | 0.727 | TRUE | 0.995 | *Lower in Lamp2KO* |
| NAD de novo biosynthesis I (from aspartate) | -0.0148 | 0.042 | -0.351 | 0.728 | FALSE | 0.995 | *Lower in Lamp2KO* |
| superpathway of branched chain amino acid biosynthesis | 0.0086 | 0.025 | 0.346 | 0.733 | FALSE | 0.995 | *Higher in Lamp2KO* |
| folate transformations III (E. coli) | -0.0091 | 0.028 | -0.331 | 0.743 | FALSE | 0.995 | *Lower in Lamp2KO* |
| glycolysis III (from glucose) | -0.0137 | 0.048 | -0.286 | 0.777 | FALSE | 0.995 | *Lower in Lamp2KO* |
| superpathway of adenosine nucleotides de novo biosynthesis I | 0.0104 | 0.044 | 0.238 | 0.813 | TRUE | 0.995 | *Higher in Lamp2KO* |
| guanosine ribonucleotides de novo biosynthesis | 0.008 | 0.036 | 0.22 | 0.827 | FALSE | 0.995 | *Higher in Lamp2KO* |
| gondoate biosynthesis (anaerobic) | -0.0096 | 0.045 | -0.215 | 0.831 | FALSE | 0.995 | *Lower in Lamp2KO* |
| L-isoleucine biosynthesis I (from threonine) | 0.0065 | 0.055 | 0.118 | 0.907 | TRUE | 0.995 | *Higher in Lamp2KO* |
| L-valine biosynthesis | 0.0065 | 0.055 | 0.118 | 0.907 | TRUE | 0.995 | *Higher in Lamp2KO* |
| pyruvate fermentation to isobutanol (engineered) | 0.0065 | 0.055 | 0.118 | 0.907 | TRUE | 0.995 | *Higher in Lamp2KO* |
| superpathway of purine nucleotides de novo biosynthesis I | -0.0041 | 0.038 | -0.107 | 0.916 | FALSE | 0.995 | *Lower in Lamp2KO* |
| chorismate biosynthesis I | 0.0044 | 0.049 | 0.09 | 0.929 | TRUE | 0.995 | *Higher in Lamp2KO* |
| chorismate biosynthesis from 3-dehydroquinate | 0.0011 | 0.029 | 0.04 | 0.969 | FALSE | 0.995 | *Higher in Lamp2KO* |
| 5-aminoimidazole ribonucleotide biosynthesis II | -0.0005 | 0.033 | -0.015 | 0.988 | FALSE | 0.995 | *Lower in Lamp2KO* |
| superpathway of 5-aminoimidazole ribonucleotide biosynthesis | -0.0005 | 0.033 | -0.015 | 0.988 | FALSE | 0.995 | *Lower in Lamp2KO* |
| inosine 5'-phosphate degradation | 0.0003 | 0.049 | 0.006 | 0.995 | FALSE | 0.995 | *Higher in Lamp2KO* |

**Supplementary Table S8. Sensitivity analysis comparing the primary analysis set (n = 48) with the full cohort including the two low-depth libraries (n = 50).** Values for diversity metrics are means for wild-type (WT) and *Lamp2KO* mice, with the genotype p-value in parentheses.

| **Metric** | **Primary analysis set (n = 48)** | **Full cohort (n = 50)** |
| --- | --- | --- |
| Samples (*Lamp2*KO, WT) | 48 (27, 21) | 50 (29, 21) |
| Cages | 27 | 28 |
| Genera tested (prevalence ≥ 10%) | 886 | 888 |
| Shannon, WT / *Lamp2*KO (genotype p) | 2.74 / 2.75 (0.524) | 2.74 / 2.76 (0.652) |
| Simpson (1 − D), WT / *Lamp2*KO (genotype p) | 0.89 / 0.89 (0.409) | 0.89 / 0.89 (0.485) |
| Observed genera, WT / *Lamp2*KO (genotype p) | 416 / 391 (0.179) | 416 / 378 (0.143) |
| PERMANOVA, genotype — Aitchison (R², p) | 0.016, 0.265 | 0.016, 0.218 |
| PERMANOVA, genotype — Bray–Curtis (R², p) | 0.015, 0.136 | 0.014, 0.235 |
| Differential abundance — genera with nominal p < 0.05 | 55 | 62 |
| Differential abundance — minimum BH-FDR q | 0.76 | 0.66 |
| Differential abundance — genera at q < 0.05 | 0 | 0 |

**Footnotes:** The primary analysis set excludes two *Lamp2*KO libraries with <3 × 10⁵ classified genus-level reads; the full cohort includes all 50 sequenced libraries. Genera were retained if present in ≥10% of samples within each set. Alpha-diversity genotype p-values are from linear mixed models (metric ~ genotype + log₁₀ depth + cage random intercept); genotype was also non-significant in the cohort-wide models reported in the main text. PERMANOVA was performed on Aitchison (centered-log-ratio, CLR) and Bray–Curtis distances with 999 permutations restricted within cage (cage-blocked) and evaluated marginally, adjusting for housing, age, and sequencing batch; the genotype term is shown (R², proportion of variance explained). Differential abundance was assessed per genus using CLR-transformed counts in linear mixed models (genotype, adjusting for housing, age, batch, log₁₀ sequencing depth, and host read fraction, with cage as a random intercept); p-values were adjusted across tested genera using the Benjamini–Hochberg false discovery rate (BH-FDR). Abbreviations: WT, wild-type; *Lamp2*KO, *Lamp2* knockout; CLR, centered-log-ratio; PERMANOVA, permutational multivariate analysis of variance; BH-FDR, Benjamini–Hochberg false discovery rate.

**Supplemental Figures:**

**Supplementary Figure S1. Per-sample taxonomic composition of fecal microbiomes in *Lamp2*KO and WT mice.**

Stacked bar plots show per-sample relative abundance of the top 10 taxa at each taxonomic rank (remaining taxa grouped as “Other”), based on One Codex relative-abundance outputs. Each bar represents one mouse fecal sample; samples are grouped by genotype, and sample IDs are shown on the x-axis.

(A) Phylum-level composition.

(B) Genus-level composition.

(C) Species-level composition.

These panels are descriptive and illustrate inter-individual variability; formal genotype association testing was performed at the genus level using cage-aware models (see Results and Supplementary Table S6).

**Supplementary Figure S2. Sample-level abundance profiles of the 50 most abundant inferred microbial metabolic pathways.**

**Heatmap of One Codex MetaCyc pathway abundances for the 50 most abundant pathways across the cohort. Rows represent pathways and columns represent individual samples; values are shown as copies per million (CPM) with the color scale indicated in the figure. Samples are grouped by genotype, and x-axis labels include age and housing metadata. Statistical testing of pathway differences between *Lamp2*KO and WT mice was performed on this pathway set using CLR-based mixed-effects models and cage-blocked PERMANOVA (see Methods and Supplementary Table S7).**

**Supplementary Figure S3. Host-read fraction is elevated in Lamp2KO mice.** Fraction of non-specific/host reads per library by genotype (n = 50; WT n = 21, Lamp2KO n = 29). Boxes show the median and interquartile range; whiskers extend to 1.5× IQR; each point is one library. The genotype difference is significant (Welch's two-sided t-test, p = 0.005; p = 0.008 in a cage-aware mixed model; Table 1).
