# Supplementary material for: Shotgun metagenomic profiling of the fecal microbiome in Lamp2 knockout mice reveals limited genotype-associated differences under standard housing
Source: PLoS One. 2026 Sep 18;21(9):e0357009. doi: 10.1371/journal.pone.0357009 (PMC13588336; doi:10.1371/journal.pone.0357009)
Supplement: Supplementary file 2 [file pone.0357009.s003.docx]

We thank the editor and Reviewer #1 for their careful evaluation. We have revised the manuscript comprehensively in response to every point, and believe the changes have substantially strengthened it.

Below we address each comment in turn under Journal Requirements, Editor Comments, and Reviewer Comments. For each, we give our response followed by the specific manuscript changes and their locations; quoted text shows the original wording followed by the revised wording. Page and line numbers refer to the revised manuscript.

**Journal Requirements:**

When submitting your revision, we need you to address these additional requirements.

1.Please ensure that your manuscript meets PLOS ONE's style requirements, including those for file naming. The PLOS ONE style templates can be found at

https://journals.plos.org/plosone/s/file?id=wjVg/PLOSOne_formatting_sample_main_body.pdf and <https://journals.plos.org/plosone/s/file?id=ba62/PLOSOne_formatting_sample_title_authors_affiliations.pdf>

**Answer:** Confirmed. The manuscript and file naming have been formatted according to PLOS ONE style requirements.

2. Thank you for stating the following financial disclosure:

“George and Marie Vergottis Postdoctoral Fellowship from Harvard Medical School awarded to Maria Emfietzoglou”

Please state what role the funders took in the study.  If the funders had no role, please state: "The funders had no role in study design, data collection and analysis, decision to publish, or preparation of the manuscript."

If this statement is not correct you must amend it as needed.

Please include this amended Role of Funder statement in your cover letter; we will change the online submission form on your behalf.

**Answer:** The funders had no role in study design, data collection and analysis, decision to publish, or preparation of the manuscript. We have included this in the cover letter.

3. Thank you for stating the following in the Competing Interests section:

“I have read the journal's policy and the authors of this manuscript have the following competing interests: Demetrios G. Vavvas is a consultant for TwentyTwenty Therapeutics, Sumitomo Pharma (Sunovion), Inhibikase, Generian, Cambridge Polymer Group, and Roche; reports honoraria from Roche, Alcon, and Bausch + Lomb; and has received expert testimony fees from Novartis. Joan W. Miller is a consultant for Sumitomo Pharma and ONL Therapeutics. This does not alter our adherence to PLOS ONE policies on sharing data and materials.”

We note that one or more of the authors are employed by a commercial company: TwentyTwenty Therapeutics, Sumitomo Pharma (Sunovion), Inhibikase, Generian, Cambridge Polymer Group, and Roche

1. Please provide an amended Funding Statement declaring this commercial affiliation, as well as a statement regarding the Role of Funders in your study. If the funding organization did not play a role in the study design, data collection and analysis, decision to publish, or preparation of the manuscript and only provided financial support in the form of authors' salaries and/or research materials, please review your statements relating to the author contributions, and ensure you have specifically and accurately indicated the role(s) that these authors had in your study. You can update author roles in the Author Contributions section of the online submission form.

Please also include the following statement within your amended Funding Statement.

“The funder provided support in the form of salaries for authors [insert relevant initials], but did not have any additional role in the study design, data collection and analysis, decision to publish, or preparation of the manuscript. The specific roles of these authors are articulated in the ‘author contributions’ section.”

If your commercial affiliation did play a role in your study, please state and explain this role within your updated Funding Statement.

**Answer:** We thank the editorial office. To clarify, no author is employed by any of the listed commercial entities; the relationships disclosed are consulting, honoraria, and expert-testimony arrangements only, and none of these entities provided funding, salary support, materials, or data for the present study. We have accordingly updated both statements as follows and included them in the cover letter for transcription into the submission form:

**Funding Statement:** This work was supported by the George and Marie Vergottis Postdoctoral Fellowship from Harvard Medical School (awarded to M.E.). The funder had no role in study design, data collection and analysis, decision to publish, or preparation of the manuscript. No author is employed by TwentyTwenty Therapeutics, Sumitomo Pharma/Sunovion, Inhibikase, Generian, Cambridge Polymer Group, Roche, ONL Therapeutics, Alcon, Bausch + Lomb, or Novartis, and none of these entities provided funding, salary support, materials, or data for the present study. The relationships with these entities are consulting, honoraria, or expert-testimony arrangements unrelated to this work, as detailed in the Competing Interests statement.

2. Please also provide an updated Competing Interests Statement declaring this commercial affiliation along with any other relevant declarations relating to employment, consultancy, patents, products in development, or marketed products, etc.

Within your Competing Interests Statement, please confirm that this commercial affiliation does not alter your adherence to all PLOS ONE policies on sharing data and materials by including the following statement: "This does not alter our adherence to  PLOS ONE policies on sharing data and materials.” (as detailed online in our guide for authors http://journals.plos.org/plosone/s/competing-interests) . If this adherence statement is not accurate and  there are restrictions on sharing of data and/or materials, please state these. Please note that we cannot proceed with consideration of your article until this information has been declared.

Please include both an updated Funding Statement and Competing Interests Statement in your cover letter. We will change the online submission form on your behalf.

**Competing Interests Statement:** Demetrios G. Vavvas is a consultant for TwentyTwenty Therapeutics, Sumitomo Pharma/Sunovion, Inhibikase, Generian, Cambridge Polymer Group, and Roche; reports honoraria from Roche, Alcon, and Bausch + Lomb; and has received expert testimony fees from Novartis. Joan W. Miller is a consultant for Sumitomo Pharma and ONL Therapeutics. These relationships are unrelated to the present work, and no author is employed by these entities. This does not alter our adherence to PLOS ONE policies on sharing data and materials.

4. Please include your full ethics statement in the ‘Methods’ section of your manuscript file. In your statement, please include the full name of the IRB or ethics committee who approved or waived your study, as well as whether or not you obtained informed written or verbal consent. If consent was waived for your study, please include this information in your statement as well.

**Answer:** We have adjusted the ethics statement in the Methods section (p. 6, lines 136-141). All animal procedures were approved by the Massachusetts General Brigham Institutional Animal Care and Use Committee (IACUC; protocol 2021N000118). All procedures adhered to the ARVO Statement for the Use of Animals in Ophthalmic and Vision Research. This study did not involve human participants or human-derived samples; therefore, institutional review board approval and informed consent were not applicable.

5. If the reviewer comments include a recommendation to cite specific previously published works, please review and evaluate these publications to determine whether they are relevant and should be cited. There is no requirement to cite these works unless the editor has indicated otherwise.

**Answer:** Not applicable; there was no such recommendation.

**Additional Editor Comments:**

- **Statistical Power & Type II Error:** Address the limited statistical power and the associated risk of a Type II error (false negatives), ensuring that the conclusion of "minimal differences" is robustly supported.

**Response:** We thank the editor for this important point and have addressed the limited statistical power and associated Type II error risk directly, in two ways: by quantifying the effect sizes the study was powered to detect, and by demonstrating that the negative result is robust to our sample-exclusion decision. *(The complementary softening of negative-result language is detailed in our response to the following comment, "Overinterpretation of Negative Results.")*

We added a simulation-based power analysis on the primary analysis set (n = 48; 80% power, two-sided α = 0.05). The cage-correlated design is the principal constraint on power: cage accounted for approximately half the variance in alpha diversity (intraclass correlation ≈ 0.48). With this structure, the study had ~80% power to detect a between-genotype difference of ≥ 0.20 in Shannon diversity (Cohen's d ≈ 0.7; observed difference 0.01); for community structure, the observed genotype effect (R² = 0.016) was below the level expected for a random two-group split (≈ 0.022), and simulation confirmed ~80% power to detect a coherent community shift exceeding this chance level; and for per-genus differential abundance, a genus of typical variance would have required a centered-log-ratio difference of ≥ ~1.25 (≈ 3.5-fold change) to be detected after Benjamini–Hochberg correction across 886 genera.

These analyses establish that the study could detect moderate-to-large genotype effects, so the conclusion of limited genotype-associated differences is supported within explicitly stated detection limits, while smaller or low-prevalence effects cannot be excluded. We further confirmed that this conclusion does not depend on excluding the two low-depth libraries (sensitivity analysis; Supplementary Table S8). The specific changes are:

1. Methods, Statistical analysis (p. 13, lines 282–290): added a new "Statistical power" subsection: "To quantify the effect sizes the study could detect, we performed a simulation-based power analysis on the primary analysis set (n = 48; 80% power, two-sided α = 0.05). For alpha diversity, genotype-associated shifts of increasing magnitude were added to Shannon values and re-tested with the cage-aware mixed model. For community structure, coherent genotype-associated shifts were injected into the CLR-transformed profiles and re-tested by cage-blocked PERMANOVA. For per-genus differential abundance, the minimum detectable CLR difference was derived from the median per-genus residual variance under Benjamini–Hochberg control across all tested genera."
2. Discussion, limitations paragraph (p. 29, lines 667–677): "Finally, because effective replication is constrained by cage structure, the study is positioned to detect moderate, consistent genotype effects but cannot exclude subtle effects below conservative detection thresholds." → "Finally, effective replication is constrained by cage structure (cage accounted for approximately half the variance in alpha diversity; intraclass correlation ≈ 0.48), which limits statistical power. By simulation, the study had approximately 80% power to detect a between-genotype difference of ≥ 0.20 in Shannon diversity (Cohen's d ≈ 0.7; observed difference 0.01) and a coherent community-level shift exceeding the variance expected for a random grouping (observed genotype R² = 0.016, below the chance expectation of ≈ 0.022); for per-genus differential abundance, a genus of typical variance would have required a ≈ 3.5-fold difference (centered-log-ratio ≥ 1.25) to be detected after correction across 886 genera. The study was therefore positioned to detect moderate-to-large genotype effects but cannot exclude smaller or low-prevalence effects, and the negative findings should be interpreted as no detectable effect above these magnitudes."
3. Supplementary information: added Supplementary Table S8 (sensitivity analysis demonstrating the negative result is unchanged when the two low-depth libraries are included; detailed in our response to Reviewer #1, Comment 1).

- **Overinterpretation of Negative Results:** Soften statements that definitively conclude an absence of biological effect, framing them instead within the limits of the current sample size and study design.

**Response:** We thank the editor for this important point. We have revised the manuscript throughout to frame our negative findings as a failure to detect effects given the current sample size and study design, rather than as definitive evidence of no biological effect, replacing absolute formulations with detection-based language and attaching the relevant power limitation directly to each claim. The specific changes are:

1. Title (p. 1, line 1-2): "shows minimal genotype-associated differences" → "reveals limited genotype-associated differences under standard housing."
2. Abstract, Conclusions (p. 3, lines 54–58): "Lamp2 deficiency was not associated with robust, cage-independent shifts…" → "we did not detect robust, cage-independent shifts… associated with Lamp2 deficiency…; given the sample size, smaller or compartment-specific effects cannot be excluded."
3. Results, alpha diversity (p. 15-16, lines 341–344): "these data indicate that Lamp2 deficiency is not associated with measurable differences in within-sample genus diversity…" → "these data indicate that we did not detect genotype-associated differences in within-sample genus diversity…, although the available sample size provides limited power to detect small effects."
4. Results, differential abundance (p. 21, lines 435–438): added to the closing sentence: "…in this cohort, while recognizing that the cohort size limits power to detect small or low-prevalence effects."
5. Discussion, paragraph 2 (p. 24, lines 525–527): "Our data indicate that Lamp2 deficiency is not a major determinant of fecal microbiome structure…" → "Within the limits of this cohort's sample size and design, our data indicate that we did not detect a major effect of Lamp2 deficiency on fecal microbiome structure…"
6. Discussion, retinal-phenotype paragraph (p. 27, lines 612–613): "These data therefore argue against large baseline shifts… as a primary driver…" → "These data therefore suggest that large baseline shifts… are unlikely to be a primary driver…"
7. Conclusions (p. 29-30, lines 679–687): "Lamp2 deficiency in mice does not produce robust, reproducible shifts…" → "we did not detect robust, reproducible shifts… attributable to Lamp2 deficiency…; the cohort size and single-time-point, feces-based design constrain the effect magnitudes that can be excluded."

- **Biological & Functional Validation:** Supplement the predictive functional pathway inferences with actual biological validation, or clearly outline this limitation.

**Response:** We thank the editor. Because our functional data derive from gene-content–based pathway inference (functional potential) rather than measured activity, and the single-time-point fecal design precludes adding orthogonal assays to the existing samples, we have chosen to outline this limitation explicitly. The specific changes are:

1. Discussion, functional paragraph (p. 25, lines 559–570): replaced the final sentence with: "Importantly, these pathway profiles represent inferred functional potential derived from microbial gene content rather than measured functional activity: shotgun metagenomics quantifies the relative abundance of genes assigned to annotated pathways but does not capture gene expression, enzyme activity, or metabolite production. We did not perform orthogonal biological validation (e.g., metatranscriptomics, targeted metabolomics, or biochemical assays), so the exploratory dTDP-β-L-rhamnose association should be regarded as a hypothesis to be tested directly—for example, by quantifying rhamnose-containing cell-envelope glycans or the corresponding nucleotide-sugar intermediates—rather than as evidence of an altered functional output in vivo. As with all reference-based functional inference from metagenomes, pathway-level results additionally reflect relative-abundance patterns conditioned on database annotation and should be interpreted in that context [13,16]."
2. Discussion, Limitations paragraph (p. 28, lines 650–652): inserted after "…reference-based inference for taxonomy and pathway annotation [16,17]": "Critically, all functional results reflect predicted pathway potential inferred from metagenomic gene content and require direct biological validation before mechanistic interpretation."
3. Results heading (p. 23, lines 503-504): "Functional pathway profiles show minimal genotype-associated differences" → "Inferred functional pathway profiles show minimal genotype-associated differences."

- **Methodological Rigor:** Ensure that potential confounding from host-derived reads, batch effects, and potential low-level contamination (especially for exploratory taxa findings) are thoroughly assessed and addressed.

**Response:** We thank the editor and address the three sources of potential confounding in turn.

(i) Host-derived reads. We examined the genotype difference in host-read fraction in detail and found it reflects bona fide Mus musculus reads (7.5% vs 3.5% of total reads; p = 0.004), remains significant after accounting for cage (p = 0.008), and is biologically plausible given LAMP2's role in lysosomal/autophagic turnover. Critically, it does not confound the microbial analysis: host read fraction was included as a covariate in all genus- and pathway-level models, and the differential-abundance conclusion was unchanged when it was omitted (no genera FDR-significant either way). This is detailed in our response to Reviewer #1, Comment 2, with corresponding Methods, Results, and Discussion revisions.

(ii) Batch effects. Sequencing batch was included as a fixed covariate in all alpha-diversity, differential-abundance, and PERMANOVA models. Both genotypes were represented across all five sequencing batches, so genotype is not confounded with batch. In the marginal, cage-blocked PERMANOVA, batch was the largest non-residual term but was not statistically significant (R² = 0.10, p = 0.115), and genotype remained non-significant in the same model (R² = 0.016, p = 0.265; Table 3); thus batch variation is modeled and does not alter the genotype conclusion. We have made the non-confounding explicit in the Results.

(iii) Low-level contamination of exploratory taxa. We agree the exploratory paired-cage genera warrant a contamination assessment. Because this dataset does not include negative (blank) controls or per-sample DNA quantitation, formal decontam-based classification was not applicable; we therefore assessed contamination using three complementary criteria: cross-referencing against published reagent/laboratory contaminant lists (Salter et al. [14]), absolute abundance, and a frequency-based diagnostic testing whether each genus was relatively enriched in lower-biomass samples (relative abundance vs sequencing depth). Six of the seven exploratory genera (Pseudomonas, Stenotrophomonas, Halomonas, Herbaspirillum, Streptomyces, and Veillonella) are recognized environmental or reagent contaminants; all seven were present at trace relative abundance (≤ 0.003%), and several showed the negative abundance–depth relationship characteristic of contaminants. Only Thomasclavelia, a recognized gut commensal, is not a typical contaminant. None of these genera reached significance in the cohort-wide, cage-aware models, consistent with their being low-abundance, exploratory signals rather than robust genotype effects. We now state this explicitly and flag these taxa as likely environmental/reagent contaminants. The specific changes are:

1. Methods, Paired-cage analysis (p. 11, lines 250–255) appended: "Genera highlighted by this exploratory analysis were additionally screened for laboratory or reagent contamination by cross-referencing against published common-contaminant genera [14], examining their relative abundance, and testing whether their relative abundance was inversely associated with sequencing depth (a biomass-based frequency diagnostic); formal decontam-based classification [15] was not applied because the dataset did not include negative (blank) controls or DNA quantitation."
2. Results, beta diversity (p. 19, lines 374–377): appended "Both genotypes were represented across all five sequencing batches, so genotype was not confounded with batch; batch was modeled as a covariate and, although it was the largest non-residual term, it was not significant (Table 3)."
3. Results, paired-cage analysis (p. 22, lines 462–467): appended "Consistent with a technical origin, six of the seven genera (Halomonas, Stenotrophomonas, Veillonella, Pseudomonas, Herbaspirillum, and Streptomyces) are recognized environmental or reagent contaminants [14], all were present at trace relative abundance (≤0.003%), and several were relatively enriched in lower-biomass samples (a contamination-consistent pattern); only Thomasclavelia, a gut commensal, is not a typical contaminant."
4. Discussion, taxon-level paragraph (p. 24, lines 537–543): revised the existing caveat to "Indeed, six of the seven taxa implicated in the paired analysis are recognized environmental or reagent contaminants [14], all were present at trace relative abundance (≤0.003%), and several showed the inverse abundance–depth relationship characteristic of contamination; only Thomasclavelia is a typical gut commensal. These signals are therefore most consistent with low-level technical background rather than genotype-driven biology, and independent replication and targeted validation would be required before assigning biological significance to these taxa-level trends [14,15]."
5. Supplementary Table S6 (p. 8): added a footnote "Six of the seven genera (all except Thomasclavelia) are recognized environmental or reagent contaminants (Salter et al. [14]) and are present at trace relative abundance (≤0.003%); none reached significance in the primary cohort-wide models."

- **Study Design Variables:** Account for potential confounding from undercharacterized environmental/dietary variables, the lack of sex-based analysis, and the limitations of relying on a single time-point fecal sampling strategy rather than a longitudinal design.

**Response:** We thank the editor for these design considerations and address each in turn.

(i) Environmental/dietary variables. All animals received an identical diet and water source ad libitum within a single SPF facility, were generated as genotype-discordant littermates from common heterozygous dams, were sampled at a standardized time of day, and were analyzed with cage modeled as a random effect—so the principal sources of environmental variation in murine microbiome studies were controlled by design rather than left to vary. We have made this explicit and acknowledged the residual variation that was not individually recorded. The following text was added to the Discussion (p. 28, lines 641–645): "Environmental variation, a dominant driver of murine microbiome composition, was minimized by design: control and knockout animals were generated as littermates from heterozygous dams, housed in a single SPF facility on a common diet and water source provided ad libitum, sampled at a standardized time of day, and analyzed with cage modeled as a random effect. However, residual unmeasured environmental variation cannot be fully excluded."

(ii) Sex-based analysis. Because Lamp2 is X-linked, only hemizygous males are uniformly null; heterozygous females are X-inactivation mosaics with variable penetrance, mirroring the more severe and uniform presentation of male patients with Danon disease. The male-only design was therefore a deliberate choice to model a uniform loss-of-function genotype, not an oversight. We now state this rationale in the Methods (p. 6, lines 127–132): "Because Lamp2 is X-linked, hemizygous males (Lamp2−/Y) are uniformly null, whereas heterozygous females (Lamp2+/−) are mosaic for X-inactivation and show variable phenotypic penetrance, paralleling the more severe and uniform presentation of male patients with Danon disease. We therefore studied only male mice to model a uniform loss-of-function genotype; sex-based and sex-by-genotype comparisons were consequently not possible by design." We also acknowledge the corresponding limitation in the Discussion (p. 28, lines 653–655): "Because Lamp2 is X-linked and the study was, by design, restricted to hemizygous males to obtain a uniform null genotype, our findings may not generalize to heterozygous female carriers, and sex-specific or sex-by-genotype microbiome effects could not be evaluated."

(iii) Single time-point vs. longitudinal design. We agree this is a limitation and now state it explicitly, while noting that the cohort nonetheless provides cross-sectional coverage across four ages. The following was added to the Discussion (p. 28-29, lines 655–666): "Fecal sampling was also cross-sectional: although the cohort spanned four ages (3–12 months), each animal contributed a single sample rather than repeated longitudinal measurements. We therefore captured an age-stratified snapshot of community structure but could not assess within-animal temporal stability, individual trajectories, or age-by-genotype dynamics; a longitudinal design with repeated sampling of the same animals would be required to detect transient or time-dependent genotype effects."

We believe these additions transparently account for the design considerations raised.

- Please add more publication level figures.

**Response:** We thank the editor. We have expanded and upgraded the figure set to publication standard: the manuscript now has four main figures (two new, two regenerated) plus one new supplementary figure. Because figures are numbered by order of appearance, the two original figures were renumbered (original Fig 1 → Fig 2; original Fig 2 → Fig 4), and all callouts and legends were updated. The figures are:

1. Figure 1 (new): genus-level alpha diversity.

2. Figure 2 (regenerated; formerly Fig 1): Aitchison PCoA.

3. Figure 3 (new): differential-abundance volcano.

4. Figure 4 (regenerated; formerly Fig 2): taxonomic composition.

5. Supplementary Figure S3 (new): host-read fraction by genotype.

- Manuscript needs to be rewritten as per the importance of shotgun metagenomics.

**Response:** We thank the editor for this guidance and have revised the manuscript to foreground the rationale for, and analytical value of, shotgun metagenomics across the Abstract, Introduction, Methods, and Discussion.

1. Introduction. We added a dedicated paragraph motivating the choice of method (p. 5, lines 99–109): "We addressed this question using shotgun metagenomic sequencing rather than marker-gene (16S rRNA) profiling. Whereas amplicon approaches survey a single marker gene—typically limiting resolution to the genus level and introducing primer and 16S copy-number biases—shotgun metagenomics sequences total community DNA, supporting higher taxonomic resolution (to the species level in this study) and, importantly, direct inference of microbial functional potential from gene content [13]. This added resolution and functional dimension make shotgun metagenomics well suited to detecting subtle, host-genotype–associated shifts in both community composition and metabolic capacity, and they raise the evidentiary bar for concluding such shifts are absent: a higher-resolution, function-aware method that nonetheless detects minimal differences provides stronger evidence against a robust microbiome effect than a lower-resolution survey would."
2. Methods. We clarified why genus-level tables were used for inference while preserving the higher-resolution data shotgun provides (p. 8, lines 172–175): "Although shotgun profiling can resolve taxa below the genus level (e.g., to the species level), genus-level tables were used for inferential analyses to reduce sparsity and classifier-level misassignment, which are pronounced for low-biomass features in fecal shotgun data and which inflate variance in differential-abundance testing."
3. Discussion. We now articulate the analytical strength of the method and how it bears on a negative finding (p. 28, lines 634–639): "Foremost is the use of shotgun metagenomics, which provides higher taxonomic resolution (to the species level here) and direct functional-potential profiling, increasing sensitivity to both compositional and metabolic genotype effects; that a method of this resolution detected no robust, cage-independent shift strengthens the inference that Lamp2 loss does not substantially remodel the bulk fecal community."
4. Abstract. We revised the Background (p. 2, lines 30-31) to make the methodological framing explicit, noting that the question had not been systematically evaluated "using methods that profile both taxonomic composition and microbial functional potential, such as shotgun metagenomics."

**Reviewer comments**

Reviewer #1: I have concerns about technical rigor.

1. The exclusion of two low-depth samples lacks sensitivity analysis (they claim similar results but show nothing).

**Response:** We thank the reviewer for this point and agree that the prior statement was not adequately supported. We have now performed a formal sensitivity analysis repeating the complete genus-level pipeline, including alpha diversity, beta diversity (cage-blocked PERMANOVA), and per-genus differential abundance, on the full cohort including the two low-depth libraries (n = 50), and compared it directly with the primary analysis set (n = 48). The results are concordant across both sets: within-sample diversity (Shannon, Simpson, observed genera) was not significantly associated with genotype, community structure was not significantly associated with genotype under either Aitchison or Bray–Curtis distances, and no genus reached Benjamini–Hochberg significance in either set (minimum q = 0.76 and 0.66 for the primary and full sets, respectively). These results are presented in new Supplementary Table S8. The specific changes are:

1. Methods, sequence quality control (p. 9, lines 194–197): "Sensitivity analyses including all samples yielded similar results." → "To confirm that excluding these two libraries did not influence our conclusions, we repeated all genus-level analyses on the full cohort (n = 50); results were concordant with the primary analysis set and are reported in Supplementary Table S8."
2. Results, differential abundance (p. 21, lines 438–439): added "These conclusions were unchanged in a sensitivity analysis including the two excluded low-depth libraries (n = 50; Supplementary Table S8)."
3. Supplementary information (p. 12-13): added Supplementary Table S8 (sensitivity analysis, primary vs full cohort).

2. The host read fraction differs significantly by genotype (8.5% vs 4.4%, p=0.005) and may be biological, not just technical.

Response: We thank the reviewer for this insightful observation and agree it should not be dismissed as purely technical. We examined the host signal more closely and confirm it is driven by bona fide Mus musculus reads rather than generic non-specific mapping: the mouse-specific read fraction was higher in Lamp2KO than WT mice (7.5% vs 3.5% of total reads across the cohort; p = 0.004), paralleling the non-specific host fraction the reviewer notes (8.5% vs 4.4%). This difference persisted after accounting for cage as a random effect (genotype p = 0.008). We now acknowledge that an elevated fecal host-DNA fraction in Lamp2KO mice is biologically plausible: LAMP2 is essential for lysosomal function and autophagy, and impaired autophagic turnover in the intestinal epithelium could increase epithelial cell shedding and thus host DNA recovered in feces. We cannot, however, distinguish this from technical sources in the present dataset, and we therefore present it transparently as a potentially biological signal warranting dedicated study rather than over-interpreting it. Importantly, this difference does not confound our microbial findings: the host read fraction was included as a covariate in all genus- and pathway-level differential-abundance models, and re-running the genus-level analysis with and without this covariate yielded the same conclusion (no genera significant after FDR correction in either case). The specific changes are:

1. Methods, Quality control and primary analysis set (p. 8-9, lines 188–191): "'Host/non-specific' reads denote sequences assigned to host genomes or to non-unique microbial classifications by the pipeline; this metric was treated as a technical characteristic rather than a biological endpoint." → ““Host/non-specific” reads denote sequences assigned to host genomes or to non-unique microbial classifications by the pipeline. We included this metric as a covariate in all community analyses and additionally examined it as a candidate biological signal (see Results and Discussion).”
2. Results, Cohort design and sequencing metrics (p. 14, lines 308–314): "The host/non-specific read fraction was higher in Lamp2KO than WT (8.5% ± 6.8% vs 4.4% ± 2.6%; p = 0.005); this metric was treated as a technical characteristic, and downstream community analyses were performed using compositional approaches and covariate adjustment as described in Methods." → "The host/non-specific read fraction was higher in Lamp2KO than WT (8.5% ± 6.8% vs 4.4% ± 2.6%; p = 0.005). This signal reflected bona fide Mus musculus reads (mouse-specific fraction 7.5% vs 3.5% of total reads; p = 0.004) and remained significant after accounting for cage (p = 0.008). The host read fraction was included as a covariate in all downstream community analyses, which used compositional approaches as described in Methods; its potential biological interpretation is considered in the Discussion."
3. Discussion (p. 26, lines 584–591): replaced the host-reads passage with: "Notably, we observed a higher fecal host-DNA (Mus musculus) fraction in Lamp2KO mice, which persisted after accounting for cage. Because LAMP2 is required for lysosomal function and autophagy, this may reflect increased intestinal epithelial turnover or shedding in knockout animals; however, technical contributions—such as differences in stool composition or sample handling—cannot be excluded, and the observation should be regarded as hypothesis-generating and warranting direct measurement in future work. The host read fraction was included as a covariate in all differential-abundance models, and our microbial conclusions were unchanged when it was omitted."

3. Functional analysis is limited to the top 50 pathways arbitrarily.

**Response:** We thank the reviewer and have both clarified the basis for this choice and confirmed that it does not affect our conclusions. The 50-pathway set was not arbitrary: these are the most abundant and most stably quantified pathways and constitute the standard functional output returned by the analysis platform (One Codex, used by our sequencing provider Transnetyx), so focusing on them targets well-measured features and limits multiple testing. To confirm that this focus did not overlook a genotype-associated pathway, we additionally analyzed all detected pathways (n = 214) using the identical cage-aware mixed-effects model. Of the 214 pathways tested, 25 reached nominal significance (p < 0.05) but none was significant after Benjamini–Hochberg correction (minimum q = 0.23); notably, the pathway with the smallest q-value was one not included in the original 50, confirming that the cutoff did not conceal a stronger candidate. This is consistent with the primary analysis, in which the only exploratory signal — dTDP-β-L-rhamnose biosynthesis — was itself not significant after correction. The complete per-pathway results for all 214 pathways (effect sizes, p- and q-values) are provided as a table in the deposited project data. Extending the analysis to all detected pathways therefore revealed no additional genotype-associated pathway and reinforced that this exploratory signal is a modest, non-significant observation rather than a robust effect. The specific changes are:

1. Methods, Functional analysis (p. 12, lines 266–271): we revised the cohort-level analysis sentence from "For cohort-level comparisons, analyses were restricted to the 50 most abundant pathways across the cohort to focus on stable features and limit multiple testing." to "For cohort-level comparisons, we analyzed the 50 most abundant pathways across the cohort — the most stably quantified features and the standard set returned by the platform's functional export — to focus on well-measured pathways and limit multiple testing. To confirm this focus did not omit genotype-associated pathways, we additionally analyzed all detected pathways (n = 214; CLR-transformed pathway abundances) using the same cage-aware mixed-effects model; complete per-pathway results are provided in the deposited project data."
2. Results, functional (p. 23, lines 513–515): we appended "Extending the per-pathway analysis to all detected pathways (n = 214) yielded no pathway significant after Benjamini–Hochberg correction (minimum q = 0.23), confirming that focusing on the most abundant pathways did not omit a genotype-associated pathway."
3. Data Availability: the complete per-pathway differential-abundance results for all 214 detected pathways were added to the deposited Zenodo dataset, and the Data Availability statement was updated to cite the concept DOI, which always resolves to the latest version: https://doi.org/10.5281/zenodo.19112073

4. No power calculation is provided, so the negative conclusion is overstated.

**Response:** We agree and have added a simulation-based power analysis estimating the effect sizes detectable at 80% power on the observed data (new Methods subsection "Statistical power"; results reported in the Discussion). The cage-correlated design is the principal constraint on power (cage accounted for ~half the variance in alpha diversity; intraclass correlation ≈ 0.48). With this structure, the study had ~80% power to detect a between-genotype difference of ≥ 0.20 in Shannon diversity (Cohen's d ≈ 0.7; observed difference 0.01); for community structure, the observed genotype effect (R² = 0.016) was below the level expected for a random two-group split (≈ 0.022), and simulation confirmed ~80% power to detect a coherent community shift exceeding this chance level; and for per-genus differential abundance, a genus of typical variance would have required a centered-log-ratio difference of ≥ ~1.25 (≈ 3.5-fold change) to be detected after Benjamini–Hochberg correction across 886 genera. We now state explicitly that these analyses exclude moderate-to-large genotype-associated effects but cannot exclude smaller or low-prevalence differences, and the negative findings are framed throughout as "no detectable effect above these magnitudes." The specific changes are:

1. Methods, Statistical analysis (p. 13, lines 282–290): added a new "Statistical power" subsection: "To quantify the effect sizes the study could detect, we performed a simulation-based power analysis on the primary analysis set (n = 48; 80% power, two-sided α = 0.05). For alpha diversity, genotype-associated shifts of increasing magnitude were added to Shannon values and re-tested with the cage-aware mixed model. For community structure, coherent genotype-associated shifts were injected into the CLR-transformed profiles and re-tested by cage-blocked PERMANOVA. For per-genus differential abundance, the minimum detectable CLR difference was derived from the median per-genus residual variance under Benjamini–Hochberg control across all tested genera."
2. Discussion, limitations paragraph (p. 29, lines 667–677): "Finally, because effective replication is constrained by cage structure, the study is positioned to detect moderate, consistent genotype effects but cannot exclude subtle effects below conservative detection thresholds." → "Finally, effective replication is constrained by cage structure (cage accounted for approximately half the variance in alpha diversity; intraclass correlation ≈ 0.48), which limits statistical power. By simulation, the study had approximately 80% power to detect a between-genotype difference of ≥ 0.20 in Shannon diversity (Cohen's d ≈ 0.7; observed difference 0.01) and a coherent community-level shift exceeding the variance expected for a random grouping (observed genotype R² = 0.016, below the chance expectation of ≈ 0.022); for per-genus differential abundance, a genus of typical variance would have required a ≈ 3.5-fold difference (centered-log-ratio ≥ 1.25) to be detected after correction across 886 genera. The study was therefore positioned to detect moderate-to-large genotype effects but cannot exclude smaller or low-prevalence effects, and the negative findings should be interpreted as no detectable effect above these magnitudes."

5. Language is intelligible but has many errors (missing spaces after colons, backslashes in Lamp2, inconsistent hyphens, corrupted numbers on page 40).

**Response:** We thank the reviewer for prompting a careful language and formatting check. We have proofread the entire manuscript, reviewing each of the categories noted such as spacing around punctuation, rendering of the gene symbol Lamp2, hyphen and dash usage, and numerical formatting, and corrected the issues identified while ensuring consistency throughout. Specifically, we standardized the notation for the coefficient of determination to "R²" across the Results, Table 3, and the Figure 2 legend, and inserted a missing space before an in-text citation in the Introduction. We confirmed that Lamp2/LAMP2 nomenclature is applied consistently (gene italic, protein roman), that hyphen and en-dash usage is uniform (e.g., cage-aware, non-specific; host–microbe, Bray–Curtis), and that powers of ten are correctly formatted as superscripts (e.g., 3 × 10⁵) throughout.

I recommend major revision.
