# Supplementary material for: Shotgun metagenomic profiling of the fecal microbiome in Lamp2 knockout mice reveals limited genotype-associated differences under standard housing
Source: PLoS One. 2026 Sep 18;21(9):e0357009. doi: 10.1371/journal.pone.0357009 (PMC13588336; doi:10.1371/journal.pone.0357009)
Supplement: Peer review history

# Peer review: Decision Letter 0

**DOI:** 10.1371/journal.pone.0357009.r001

**Authors:** Rajesh Singh Rathore

26 May 2026

Dear Dr.  Vavvas,

Thank you for submitting your manuscript to PLOS ONE. After careful consideration, we feel that it has merit but does not fully meet PLOS ONE’s publication criteria as it currently stands. Therefore, we invite you to submit a revised version of the manuscript that addresses the points raised during the review process.

Please submit your revised manuscript by Jul 10 2026 11:59PM. If you will need more time than this to complete your revisions, please reply to this message or contact the journal office at plosone@plos.org. When you're ready to submit your revision, log on to https://www.editorialmanager.com/pone/ and select the 'Submissions Needing Revision' folder to locate your manuscript file.

Please include the following items when submitting your revised manuscript:

- A letter that responds to each point raised by the academic editor and reviewer(s). You should upload this letter as a separate file labeled 'Response to Reviewers'.
- A marked-up copy of your manuscript that highlights changes made to the original version. You should upload this as a separate file labeled 'Revised Manuscript with Track Changes'.
- An unmarked version of your revised paper without tracked changes. You should upload this as a separate file labeled 'Manuscript'.

If you would like to make changes to your financial disclosure, please include your updated statement in your cover letter. Guidelines for resubmitting your figure files are available below the reviewer comments at the end of this letter.

If applicable, we recommend that you deposit your laboratory protocols in protocols.io to enhance the reproducibility of your results. Protocols.io assigns your protocol its own identifier (DOI) so that it can be cited independently in the future. For instructions see: https://journals.plos.org/plosone/s/submission-guidelines#loc-laboratory-protocols. Additionally, PLOS ONE offers an option for publishing peer-reviewed Lab Protocol articles, which describe protocols hosted on protocols.io. Read more information on sharing protocols at https://plos.org/protocols?utm_medium=editorial-email&utm_source=authorletters&utm_campaign=protocols.

As the corresponding author, your ORCID iD is verified in the submission system and will appear in the published article. PLOS supports the use of ORCID, and we encourage all coauthors to register for an ORCID iD and use it as well. Please encourage your coauthors to verify their ORCID iD within the submission system before final acceptance, as unverified ORCID iDs will not appear in the published article. *Only*  the individual author can complete the verification step; PLOS staff *cannot*  verify ORCID iDs on behalf of authors.

We look forward to receiving your revised manuscript.

Kind regards,

Rajesh Singh Rathore, Ph.D

Academic Editor

PLOS One

Journal Requirements:

When submitting your revision, we need you to address these additional requirements.

1.Please ensure that your manuscript meets PLOS ONE's style requirements, including those for file naming. The PLOS ONE style templates can be found at

https://journals.plos.org/plosone/s/file?id=wjVg/PLOSOne_formatting_sample_main_body.pdf and

https://journals.plos.org/plosone/s/file?id=ba62/PLOSOne_formatting_sample_title_authors_affiliations.pdf

2. Thank you for stating the following financial disclosure:

“George and Marie Vergottis Postdoctoral Fellowship from Harvard Medical School awarded to Maria Emfietzoglou”

Please state what role the funders took in the study.  If the funders had no role, please state: "The funders had no role in study design, data collection and analysis, decision to publish, or preparation of the manuscript."

If this statement is not correct you must amend it as needed.

Please include this amended Role of Funder statement in your cover letter; we will change the online submission form on your behalf.

3. Thank you for stating the following in the Competing Interests section:

“I have read the journal's policy and the authors of this manuscript have the following competing interests: Demetrios G. Vavvas is a consultant for TwentyTwenty Therapeutics, Sumitomo Pharma (Sunovion), Inhibikase, Generian, Cambridge Polymer Group, and Roche; reports honoraria from Roche, Alcon, and Bausch + Lomb; and has received expert testimony fees from Novartis. Joan W. Miller is a consultant for Sumitomo Pharma and ONL Therapeutics. This does not alter our adherence to PLOS ONE policies on sharing data and materials.”

We note that one or more of the authors are employed by a commercial company: TwentyTwenty Therapeutics, Sumitomo Pharma (Sunovion), Inhibikase, Generian, Cambridge Polymer Group, and Roche

1. Please provide an amended Funding Statement declaring this commercial affiliation, as well as a statement regarding the Role of Funders in your study. If the funding organization did not play a role in the study design, data collection and analysis, decision to publish, or preparation of the manuscript and only provided financial support in the form of authors' salaries and/or research materials, please review your statements relating to the author contributions, and ensure you have specifically and accurately indicated the role(s) that these authors had in your study. You can update author roles in the Author Contributions section of the online submission form.

Please also include the following statement within your amended Funding Statement.

“The funder provided support in the form of salaries for authors [insert relevant initials], but did not have any additional role in the study design, data collection and analysis, decision to publish, or preparation of the manuscript. The specific roles of these authors are articulated in the ‘author contributions’ section.”

If your commercial affiliation did play a role in your study, please state and explain this role within your updated Funding Statement.

2. Please also provide an updated Competing Interests Statement declaring this commercial affiliation along with any other relevant declarations relating to employment, consultancy, patents, products in development, or marketed products, etc.

Within your Competing Interests Statement, please confirm that this commercial affiliation does not alter your adherence to all PLOS ONE policies on sharing data and materials by including the following statement: "This does not alter our adherence to  PLOS ONE policies on sharing data and materials.” (as detailed online in our guide for authors http://journals.plos.org/plosone/s/competing-interests) . If this adherence statement is not accurate and  there are restrictions on sharing of data and/or materials, please state these. Please note that we cannot proceed with consideration of your article until this information has been declared.

Please include both an updated Funding Statement and Competing Interests Statement in your cover letter. We will change the online submission form on your behalf.

4. Please include your full ethics statement in the ‘Methods’ section of your manuscript file. In your statement, please include the full name of the IRB or ethics committee who approved or waived your study, as well as whether or not you obtained informed written or verbal consent. If consent was waived for your study, please include this information in your statement as well.

5. If the reviewer comments include a recommendation to cite specific previously published works, please review and evaluate these publications to determine whether they are relevant and should be cited. There is no requirement to cite these works unless the editor has indicated otherwise.

Additional Editor Comments:

Dear Dr. Vavvas,

I am writing to provide a summary of the primary areas requiring attention regarding your manuscript, "Shotgun metagenomic profiling of the fecal microbiome in Lamp2 knockout mice shows minimal genotype-associated differences."

To strengthen the impact and rigor of the study, please address the following key points in your revision:

Statistical Power & Type II Error: Address the limited statistical power and the associated risk of a Type II error (false negatives), ensuring that the conclusion of "minimal differences" is robustly supported.

Overinterpretation of Negative Results: Soften statements that definitively conclude an absence of biological effect, framing them instead within the limits of the current sample size and study design.

Need more figures.

Need proper writen manucript.

Biological & Functional Validation: Supplement the predictive functional pathway inferences with actual biological validation, or clearly outline this limitation.

Methodological Rigor: Ensure that potential confounding from host-derived reads, batch effects, and potential low-level contamination (especially for exploratory taxa findings) are thoroughly assessed and addressed.

Study Design Variables: Account for potential confounding from undercharacterized environmental/dietary variables, the lack of sex-based analysis, and the limitations of relying on a single time-point fecal sampling strategy rather than a longitudinal design.

Addressing these points will significantly improve the translational relevance and methodological clarity of your work.

--

I am writing to provide a summary of the primary areas requiring attention regarding your manuscript, *"Shotgun metagenomic profiling of the fecal microbiome in Lamp2 knockout mice shows minimal genotype-associated differences."*

To strengthen the impact and rigor of the study, please address the following key points in your revision:

- **Statistical Power & Type II Error:**  Address the limited statistical power and the associated risk of a Type II error (false negatives), ensuring that the conclusion of "minimal differences" is robustly supported.
- **Overinterpretation of Negative Results:**  Soften statements that definitively conclude an absence of biological effect, framing them instead within the limits of the current sample size and study design.
- **Biological & Functional Validation:**  Supplement the predictive functional pathway inferences with actual biological validation, or clearly outline this limitation.
- **Methodological Rigor:**  Ensure that potential confounding from host-derived reads, batch effects, and potential low-level contamination (especially for exploratory taxa findings) are thoroughly assessed and addressed.
- **Study Design Variables:**  Account for potential confounding from undercharacterized environmental/dietary variables, the lack of sex-based analysis, and the limitations of relying on a single time-point fecal sampling strategy rather than a longitudinal design.
- Please add more publicaiotn level figures.
- Manuscript needs to rewrittien as per the importance of shotgun metagenomics.

Addressing these points will significantly improve the translational relevance and methodological clarity of your work.

Best regards,

Dr. Rathore

[Note: HTML markup is below. Please do not edit.]

Reviewers' comments:

Reviewer's Responses to Questions

**Comments to the Author**

1. Is the manuscript technically sound, and do the data support the conclusions?

Reviewer #1: Partly

**********

2. Has the statistical analysis been performed appropriately and rigorously?

Reviewer #1: No

**********

3. Have the authors made all data underlying the findings in their manuscript fully available?

The PLOS Data policy

Reviewer #1: Yes

**********

4. Is the manuscript presented in an intelligible fashion and written in standard English?

Reviewer #1: No

**********

Reviewer #1: I have concerns about technical rigor. The exclusion of two low-depth samples lacks sensitivity analysis (they claim similar results but show nothing). The host read fraction differs significantly by genotype (8.5% vs 4.4%, p=0.005) and may be biological, not just technical. Functional analysis is limited to the top 50 pathways arbitrarily. No power calculation is provided, so the negative conclusion is overstated. Language is intelligible but has many errors (missing spaces after colons, backslashes in Lamp2, inconsistent hyphens, corrupted numbers on page 40). I recommend major revision.

**********

what does this mean?). If published, this will include your full peer review and any attached files.

If you choose “no”, your identity will remain anonymous but your review may still be made public.

**Do you want your identity to be public for this peer review?**  For information about this choice, including consent withdrawal, please see our Privacy Policy

Reviewer #1: **Yes:** Grisel Fierros Romero

**********

[NOTE: If reviewer comments were submitted as an attachment file, they will be attached to this email and accessible via the submission site. Please log into your account, locate the manuscript record, and check for the action link "View Attachments". If this link does not appear, there are no attachment files.]

To ensure your figures meet our technical requirements, please review our figure guidelines: https://journals.plos.org/plosone/s/figures

You may also use PLOS’s free figure tool, NAAS, to help you prepare publication quality figures: https://journals.plos.org/plosone/s/figures#loc-tools-for-figure-preparation.

NAAS will assess whether your figures meet our technical requirements by comparing each figure against our figure specifications.

# Author comment: Author response to Decision Letter 1

**DOI:** 10.1371/journal.pone.0357009.r002

13 Jun 2026

We thank the editor and Reviewer #1 for their careful evaluation. We have revised the manuscript comprehensively in response to every point, and believe the changes have substantially strengthened it.

Below we address each comment in turn under Journal Requirements, Editor Comments, and Reviewer Comments. For each, we give our response followed by the specific manuscript changes and their locations; quoted text shows the original wording followed by the revised wording. Page and line numbers refer to the revised manuscript.

Journal Requirements:

When submitting your revision, we need you to address these additional requirements.

1.Please ensure that your manuscript meets PLOS ONE's style requirements, including those for file naming. The PLOS ONE style templates can be found at

https://journals.plos.org/plosone/s/file?id=wjVg/PLOSOne_formatting_sample_main_body.pdf and https://journals.plos.org/plosone/s/file?id=ba62/PLOSOne_formatting_sample_title_authors_affiliations.pdf

Answer: Confirmed. The manuscript and file naming have been formatted according to PLOS ONE style requirements.

2. Thank you for stating the following financial disclosure:

“George and Marie Vergottis Postdoctoral Fellowship from Harvard Medical School awarded to Maria Emfietzoglou”

Please state what role the funders took in the study. If the funders had no role, please state: "The funders had no role in study design, data collection and analysis, decision to publish, or preparation of the manuscript."

If this statement is not correct you must amend it as needed.

Please include this amended Role of Funder statement in your cover letter; we will change the online submission form on your behalf.

Answer: The funders had no role in study design, data collection and analysis, decision to publish, or preparation of the manuscript. We have included this in the cover letter.

3. Thank you for stating the following in the Competing Interests section:

“I have read the journal's policy and the authors of this manuscript have the following competing interests: Demetrios G. Vavvas is a consultant for TwentyTwenty Therapeutics, Sumitomo Pharma (Sunovion), Inhibikase, Generian, Cambridge Polymer Group, and Roche; reports honoraria from Roche, Alcon, and Bausch + Lomb; and has received expert testimony fees from Novartis. Joan W. Miller is a consultant for Sumitomo Pharma and ONL Therapeutics. This does not alter our adherence to PLOS ONE policies on sharing data and materials.”

We note that one or more of the authors are employed by a commercial company: TwentyTwenty Therapeutics, Sumitomo Pharma (Sunovion), Inhibikase, Generian, Cambridge Polymer Group, and Roche

1. Please provide an amended Funding Statement declaring this commercial affiliation, as well as a statement regarding the Role of Funders in your study. If the funding organization did not play a role in the study design, data collection and analysis, decision to publish, or preparation of the manuscript and only provided financial support in the form of authors' salaries and/or research materials, please review your statements relating to the author contributions, and ensure you have specifically and accurately indicated the role(s) that these authors had in your study. You can update author roles in the Author Contributions section of the online submission form.

Please also include the following statement within your amended Funding Statement.

“The funder provided support in the form of salaries for authors [insert relevant initials], but did not have any additional role in the study design, data collection and analysis, decision to publish, or preparation of the manuscript. The specific roles of these authors are articulated in the ‘author contributions’ section.”

If your commercial affiliation did play a role in your study, please state and explain this role within your updated Funding Statement.

Answer: We thank the editorial office. To clarify, no author is employed by any of the listed commercial entities; the relationships disclosed are consulting, honoraria, and expert-testimony arrangements only, and none of these entities provided funding, salary support, materials, or data for the present study. We have accordingly updated both statements as follows and included them in the cover letter for transcription into the submission form:

Funding Statement: This work was supported by the George and Marie Vergottis Postdoctoral Fellowship from Harvard Medical School (awarded to M.E.). The funder had no role in study design, data collection and analysis, decision to publish, or preparation of the manuscript. No author is employed by TwentyTwenty Therapeutics, Sumitomo Pharma/Sunovion, Inhibikase, Generian, Cambridge Polymer Group, Roche, ONL Therapeutics, Alcon, Bausch + Lomb, or Novartis, and none of these entities provided funding, salary support, materials, or data for the present study. The relationships with these entities are consulting, honoraria, or expert-testimony arrangements unrelated to this work, as detailed in the Competing Interests statement.

2. Please also provide an updated Competing Interests Statement declaring this commercial affiliation along with any other relevant declarations relating to employment, consultancy, patents, products in development, or marketed products, etc.

Within your Competing Interests Statement, please confirm that this commercial affiliation does not alter your adherence to all PLOS ONE policies on sharing data and materials by including the following statement: "This does not alter our adherence to PLOS ONE policies on sharing data and materials.” (as detailed online in our guide for authors http://journals.plos.org/plosone/s/competing-interests) . If this adherence statement is not accurate and there are restrictions on sharing of data and/or materials, please state these. Please note that we cannot proceed with consideration of your article until this information has been declared.

Please include both an updated Funding Statement and Competing Interests Statement in your cover letter. We will change the online submission form on your behalf.

Competing Interests Statement: Demetrios G. Vavvas is a consultant for TwentyTwenty Therapeutics, Sumitomo Pharma/Sunovion, Inhibikase, Generian, Cambridge Polymer Group, and Roche; reports honoraria from Roche, Alcon, and Bausch + Lomb; and has received expert testimony fees from Novartis. Joan W. Miller is a consultant for Sumitomo Pharma and ONL Therapeutics. These relationships are unrelated to the present work, and no author is employed by these entities. This does not alter our adherence to PLOS ONE policies on sharing data and materials.

4. Please include your full ethics statement in the ‘Methods’ section of your manuscript file. In your statement, please include the full name of the IRB or ethics committee who approved or waived your study, as well as whether or not you obtained informed written or verbal consent. If consent was waived for your study, please include this information in your statement as well.

Answer: We have adjusted the ethics statement in the Methods section (p. 6, lines 136-141). All animal procedures were approved by the Massachusetts General Brigham Institutional Animal Care and Use Committee (IACUC; protocol 2021N000118). All procedures adhered to the ARVO Statement for the Use of Animals in Ophthalmic and Vision Research. This study did not involve human participants or human-derived samples; therefore, institutional review board approval and informed consent were not applicable.

5. If the reviewer comments include a recommendation to cite specific previously published works, please review and evaluate these publications to determine whether they are relevant and should be cited. There is no requirement to cite these works unless the editor has indicated otherwise.

Answer: Not applicable; there was no such recommendation.

Additional Editor Comments:

• Statistical Power & Type II Error: Address the limited statistical power and the associated risk of a Type II error (false negatives), ensuring that the conclusion of "minimal differences" is robustly supported.

Response: We thank the editor for this important point and have addressed the limited statistical power and associated Type II error risk directly, in two ways: by quantifying the effect sizes the study was powered to detect, and by demonstrating that the negative result is robust to our sample-exclusion decision. (The complementary softening of negative-result language is detailed in our response to the following comment, "Overinterpretation of Negative Results.")

We added a simulation-based power analysis on the primary analysis set (n = 48; 80% power, two-sided α = 0.05). The cage-correlated design is the principal constraint on power: cage accounted for approximately half the variance in alpha diversity (intraclass correlation ≈ 0.48). With this structure, the study had ~80% power to detect a between-genotype difference of ≥ 0.20 in Shannon diversity (Cohen's d ≈ 0.7; observed difference 0.01); for community structure, the observed genotype effect (R² = 0.016) was below the level expected for a random two-group split (≈ 0.022), and simulation confirmed ~80% power to detect a coherent community shift exceeding this chance level; and for per-genus differential abundance, a genus of typical variance would have required a centered-log-ratio difference of ≥ ~1.25 (≈ 3.5-fold change) to be detected after Benjamini–Hochberg correction across 886 genera.

These analyses establish that the study could detect moderate-to-large genotype effects, so the conclusion of limited genotype-associated differences is supported within explicitly stated detection limits, while smaller or low-prevalence effects cannot be excluded. We further confirmed that this conclusion does not depend on excluding the two low-depth libraries (sensitivity analysis; Supplementary Table S8). The specific changes are:

1. Methods, Statistical analysis (p. 13, lines 282–290): added a new "Statistical power" subsection: "To quantify the effect sizes the study could detect, we performed a simulation-based power analysis on the primary analysis set (n = 48; 80% power, two-sided α = 0.05). For alpha diversity, genotype-associated shifts of increasing magnitude were added to Shannon values and re-tested with the cage-aware mixed model. For community structure, coherent genotype-associated shifts were injected into the CLR-transformed profiles and re-tested by cage-blocked PERMANOVA. For per-genus differential abundance, the minimum detectable CLR difference was derived from the median per-genus residual variance under Benjamini–Hochberg control across all tested genera."

2. Discussion, limitations paragraph (p. 29, lines 667–677): "Finally, because effective replication is constrained by cage structure, the study is positioned to detect moderate, consistent genotype effects but cannot exclude subtle effects below conservative detection thresholds." → "Finally, effective replication is constrained by cage structure (cage accounted for approximately half the variance in alpha diversity; intraclass correlation ≈ 0.48), which limits statistical power. By simulation, the study had approximately 80% power to detect a between-genotype difference of ≥ 0.20 in Shannon diversity (Cohen's d ≈ 0.7; observed difference 0.01) and a coherent community-level shift exceeding the variance expected for a random grouping (observed genotype R² = 0.016, below the chance expectation of ≈ 0.022); for per-genus differential abundance, a genus of typical variance would have required a ≈ 3.5-fold difference (centered-log-ratio ≥ 1.25) to be detected after correction across 886 genera. The study was therefore positioned to detect moderate-to-large genotype effects but cannot exclude smaller or low-prevalence effects, and the negative findings should be interpreted as no detectable effect above these magnitudes."

3. Supplementary information: added Supplementary Table S8 (sensitivity analysis demonstrating the negative result is unchanged when the two low-depth libraries are included; detailed in our response to Reviewer #1, Comment 1).

• Overinterpretation of Negative Results: Soften statements that definitively conclude an absence of biological effect, framing them instead within the limits of the current sample size and study design.

Response: We thank the editor for this important point. We have revised the manuscript throughout to frame our negative findings as a failure to detect effects given the current sample size and study design, rather than as definitive evidence of no biological effect, replacing absolute formulations with detection-based language and attaching the relevant power limitation directly to each claim. The specific changes are:

1. Title (p. 1, line 1-2): "shows minimal genotype-associated differences" → "reveals limited genotype-associated differences under standard housing."

2. Abstract, Conclusions (p. 3, lines 54–58): "Lamp2 deficiency was not associated with robust, cage-independent shifts…" → "we did not detect robust, cage-independent shifts… associated with Lamp2 deficiency…; given the sample size, smaller or compartment-specific effects cannot be excluded."

3. Results, alpha diversity (p. 15-16, lines 341–344): "these data indicate that Lamp2 deficiency is not associated with measurable differences in within-sample genus diversity…" → "these data indicate that we did not detect genotype-associated differences in within-sample genus diversity…, although the available sample size provides limited power to detect small effects."

4. Results, differential abundance (p. 21, lines 435–438): added to the closing sentence: "…in this cohort, while recognizing that the cohort size limits power to detect small or low-prevalence effects."

5. Discussion, paragraph 2 (p. 24, lines 525–527): "Our data indicate that Lamp2 deficiency is not a major determinant of fecal microbiome structure…" → "Within the limits of this cohort's sample size and design, our data indicate that we did not detect a major effect of Lamp2 deficiency on fecal microbiome structure…"

6. Discussion, retinal-phenotype paragraph (p. 27, lines 612–613): "These data therefore argue against large baseline shifts… as a primary driver…" → "These data therefore suggest that large baseline shifts… are unlikely to be a primary driver…"

7. Conclusions (p. 29-30, lines 679–687): "Lamp2 deficiency in mice does not produce robust, reproducible shifts…" → "we did not detect robust, reproducible shifts… attributable to Lamp2 deficiency…; the cohort size and single-time-point, feces-based design constrain the effect magnitudes that can be excluded."

• Biological & Functional Validation: Supplement the predictive functional pathway inferences with actual biological validation, or clearly outline this limitation.

Response: We thank the editor. Because our functional data derive from gene-content–based pathway inference (functional potential) rather than measured activity, and the single-time-point fecal design precludes adding orthogonal assays to the existing samples, we have chosen to outline this limitation explicitly. The specific changes are:

1. Discussion, functional paragraph (p. 25, lines 559–570): replaced the final sentence with: "Importantly, these pathway profiles represent inferred functional potential derived from microbial gene content rather than measured functional activity: shotgun metagenomics quantifies the relative abundance of genes assigned to annotated pathways but does not capture gene expression, enzyme activity, or metabolite production. We did not perform orthogonal biological validation (e.g., metatranscriptomics, targeted metabolomics, or biochemical assays), so the exploratory dTDP-β-L-rhamnose association should be regarded as a hypothesis to be tested directly—for example, by quantifying rhamnose-containing cell-envelope glycans or the corresponding nucleotide-sugar intermediates—rather than as evidence of an altered functional output in vivo. As with all reference-based functional inference from metagenomes, pathway-level results additionally reflect relative-abundance patterns conditioned on database annotation and should be interp

Attachment Submitted filename: Response to Reviewers.docx

# Peer review: Decision Letter 1

**DOI:** 10.1371/journal.pone.0357009.r003

**Authors:** Rajesh Singh Rathore

12 Aug 2026

Shotgun metagenomic profiling of the fecal microbiome in Lamp2 knockout mice reveals limited genotype-associated differences under standard housing

PONE-D-26-13800R1

Dear Dr. Demetrios G. Vavvas,

Thank you for submitting the manuscript, **“Shotgun metagenomic profiling of the fecal microbiome in Lamp2 knockout mice reveals limited genotype-associated differences under standard housing.”**

Following editorial evaluation, we are pleased to inform you that the manuscript is **accepted for publication in PLOS ONE** , subject to the following minor editorial requirements:

1. Please ensure that all **genus names are consistently italicized**  throughout the manuscript, including the instances in **lines 394–395, 398, and 451** .
2. Please provide a clear **declaration regarding the use of artificial intelligence (AI)**  in preparing or writing the manuscript, including a statement describing the authors’ responsibility for reviewing, verifying, and approving any AI-assisted content.

Please incorporate these final corrections and submit the revised files for production processing.

We’re pleased to inform you that your manuscript has been judged scientifically suitable for publication and will be formally accepted for publication once it meets all outstanding technical requirements.

Within one week, you’ll receive an e-mail detailing the required amendments. When these have been addressed, you’ll receive a formal acceptance letter and your manuscript will be scheduled for publication.

An invoice will be generated when your article is formally accepted. Please note, if your institution has a publishing partnership with PLOS and your article meets the relevant criteria, all or part of your publication costs will be covered. Please make sure your user information is up-to-date by logging into Editorial Manager at Editorial Manager® and clicking the ‘Update My Information' link at the top of the page. For questions related to billing, please contact billing support.

If your institution or institutions have a press office, please notify them about your upcoming paper to help maximize its impact. If they’ll be preparing press materials, please inform our press team as soon as possible -- no later than 48 hours after receiving the formal acceptance. Your manuscript will remain under strict press embargo until 2 pm Eastern Time on the date of publication. For more information, please contact onepress@plos.org.

Kind regards,

Rajesh Singh Rathore, Ph.D

Academic Editor

PLOS One

Additional Editor Comments (optional):

Please ensure that all genus names are consistently italicized throughout the manuscript, including the instances in lines 394–395, 398, and 451.

Please provide a clear declaration regarding the use of artificial intelligence (AI) in preparing or writing the manuscript, including a statement describing the authors’ responsibility for reviewing, verifying, and approving any AI-assisted content.

Reviewers' comments:

Reviewer's Responses to Questions

**Comments to the Author**

Reviewer #1: All comments have been addressed

Reviewer #2: (No Response)

**********

2. Is the manuscript technically sound, and do the data support the conclusions?

Reviewer #1: Yes

Reviewer #2: Yes

**********

3. Has the statistical analysis been performed appropriately and rigorously?

Reviewer #1: Yes

Reviewer #2: Yes

**********

4. Have the authors made all data underlying the findings in their manuscript fully available?

The PLOS Data policy

Reviewer #1: Yes

Reviewer #2: Yes

**********

5. Is the manuscript presented in an intelligible fashion and written in standard English?

Reviewer #1: Yes

Reviewer #2: Yes

**********

Reviewer #1: The authors have thoroughly and successfully addressed all concerns raised in the previous round of review.Consider depositing the raw FASTQ reads in a public repository such as the Sequence Read Archive (SRA) with a BioProject accession number to complement the processed data already available in Zenodo.onsider explicitly stating the exploratory hypothesis regarding the dTDP-β-L-rhamnose pathway as a specific area for future investigation, which could help guide follow-up studies.

Reviewer #2: The authors of this study have performed a comparison of the fecal microbiome of Lamp2 knockout mice and wild-type mice using shotgun metagenomics and have performed extensive statistical analyses that support their findings. Please ensure that genus names are always italicized (for example, lines 394-395, 398, 451). Although implied, no particular future directions have been explicitly stated. Although thoroughly explained, the manuscript is slightly verbose, especially in the discussion, and can be made more succinct and clearer.

**********

what does this mean?). If published, this will include your full peer review and any attached files.

If you choose “no”, your identity will remain anonymous but your review may still be made public.

**Do you want your identity to be public for this peer review?**  For information about this choice, including consent withdrawal, please see our Privacy Policy

Reviewer #1: **Yes:** Grisel Fierros Romero

Reviewer #2: No

**********

# Editor report: Acceptance letter

**DOI:** 10.1371/journal.pone.0357009.r004

**Authors:** Rajesh Singh Rathore

PONE-D-26-13800R1

PLOS One

Dear Dr. Vavvas,

I'm pleased to inform you that your manuscript has been deemed suitable for publication in PLOS One. Congratulations! Your manuscript is now being handed over to our production team.

At this stage, our production department will prepare your paper for publication. This includes ensuring the following:

* All references, tables, and figures are properly cited

* All relevant supporting information is included in the manuscript submission,

* There are no issues that prevent the paper from being properly typeset

You will receive further instructions from the production team, including instructions on how to review your proof when it is ready. Please keep in mind that we are working through a large volume of accepted articles, so please give us a few days to review your paper and let you know the next and final steps.

Lastly, if your institution or institutions have a press office, please let them know about your upcoming paper now to help maximize its impact. If they'll be preparing press materials, please inform our press team within the next 48 hours. Your manuscript will remain under strict press embargo until 2 pm Eastern Time on the date of publication. For more information, please contact onepress@plos.org.

You will receive an invoice from PLOS for your publication fee after your manuscript has reached the completed accept phase. If you receive an email requesting payment before acceptance or for any other service, this may be a phishing scheme. Learn how to identify phishing emails and protect your accounts at https://explore.plos.org/phishing.

If we can help with anything else, please email us at customercare@plos.org.

Thank you for submitting your work to PLOS One and supporting open access.

Kind regards,

PLOS ONE Editorial Office Staff

on behalf of

Dr. Rajesh Singh Rathore

Academic Editor

PLOS One
